# Quorum Sensing Inhibiting Activity of *Streptomyces coelicoflavus* Isolated from Soil

**DOI:** 10.3389/fmicb.2016.00659

**Published:** 2016-05-13

**Authors:** Ramadan Hassan, Mona I. Shaaban, Fatma M. Abdel Bar, Areej M. El-Mahdy, Shadi Shokralla

**Affiliations:** ^1^Microbiology and Immunology Department, Faculty of Pharmacy, Mansoura UniversityMansoura, Egypt; ^2^Pharmacognosy Department, Faculty of Pharmacy, Mansoura UniversityMansoura, Egypt; ^3^Biodiversity Institute of Ontario, Department of Integrative Biology, University of Guelph, GuelphON, Canada

**Keywords:** Quorum sensing inhibitor, soil *Streptomyces*, *Streptomyces coelicoflavus*, *Pseudomonas* virulence factors, 1*H*-pyrrole-2-carboxylic acid, borrelidin, behenic acid, antipathogenic

## Abstract

Quorum sensing (QS) systems communicate bacterial population and stimulate microbial pathogenesis through signaling molecules. Inhibition of QS signals potentially suppresses microbial infections. Antimicrobial properties of *Streptomyces* have been extensively studied, however, less is known about quorum sensing inhibitory (QSI) activities of *Streptomyces*. This study explored the QSI potential of *Streptomyces* isolated from soil. Sixty-five bacterial isolates were purified from soil samples with morphological characteristics of *Streptomyces*. The three isolates: S6, S12, and S17, exhibited QSI effect by screening with the reporter, *Chromobacterium violaceum*. Isolate S17 was identified as *Streptomyces coelicoflavus* by sequencing of the hypervariable regions (V1–V6) of 16S rRNA and was assigned gene bank number KJ855087. The QSI effect of the cell-free supernatant of isolate S17 was not abolished by proteinase K indicating the non-enzymatic activity of QSI components of S17. Three major compounds were isolated and identified, using spectroscopic techniques (1D, 2D NMR, and Mass spectrometry), as behenic acid (docosanoic acid), borrelidin, and 1*H*-pyrrole-2-carboxylic acid. 1*H*-pyrrole-2-carboxylic acid inhibited QS and related virulence factors of *Pseudomonas aeruginosa* PAO1 including; elastase, protease, and pyocyanin without affecting *Pseudomonas* viability. At the molecular level, 1*H*-pyrrole-2-carboxylic acid suppressed the expression of QS genes (*lasI, lasR, lasA, lasB, rhlI, rhlR, pqsA*, and *pqsR*). Moreover, QSI activity of S17 was assessed under different growth conditions and ISP2 medium supplemented with glucose 0.4% w/v and adjusted at pH 7, showed the highest QSI action. In conclusion, 1*H*-pyrrole-2-carboxylic acid, one of the major metabolites of *Streptomyces* isolate S17, inhibited QS and virulence determinants of *P. aeruginosa* PAO1. The findings of the study open the scope to exploit the *in vivo* efficacy of this active molecule as anti-pathogenic and anti-virulence of *P. aeruginosa*.

## Introduction

Multi-drug-resistant bacteria represent a major problem in antibiotic therapy so there is a necessity for the development of novel therapeutic agents (Peleg and Hooper, 2010). However, the invention of new antibiotics with a distinct mechanism of action is inefficient. It has been found that bacterial communication mechanism called quorum sensing (QS) is able to regulate different functions among bacteria through QS signaling molecules “autoinducers” (Fuqua and Greenberg, 2002). Bacterial cells can sense their inoculum size via QS signals which stimulate bacterial growth with a further increase in the signaling molecules (Dong and Zhang, 2005). Then, the produced signals stimulate the transcription and the expression of virulence genes implicated in bacterial pathogenesis (Williams, 2007). Various bacterial species including *Bacillus cereus*, *Staphylococcus aureus, Vibrio* sp., and *Pseudomonas aeruginosa* utilize QS signals in regulating their virulence factors and host infection (Rutherford and Bassler, 2012). Most Gram-negative bacteria chaired the communication signaling molecules called acyl-homoserine lactones (AHLs) (Dong and Zhang, 2005). AHL-mediated QS is the key regulator of microbial virulence factors; it stimulates enzymes secretion, pigments production, bacterial motility, biofilm assembly, and toxins release. The synthesis of AHLs among Gram-negative bacteria is under the control of the synthase gene, *luxI*, and its regulator, *luxR* (Fuqua et al., 1994; Zhang et al., 2002). In *P. aeruginosa*, the QS system is mainly composed of *lasI/R*, *rhlI/R* (Pearson et al., 1995), and *pqs* cascades (Pesci et al., 1999) which coordinates the release of protease, elastase, exotoxin A, pyocyanin, hydrogen cyanide, rhamnolipids, and lectins through AHLs and other signaling molecules (Gupta et al., 2011). The main signaling molecules elaborated by *P. aeruginosa* are 3-oxo-C12-homoserine lactone, C4-homoserine lactone and 2-heptyl-3-hydroxy-4(1*H*)-quinolone which are legends for *las*, *rhl*, and *pqs* circuits, respectively (Fuqua et al., 2001).

Inhibition of the QS system could assist in the termination of the bacterial resistance, without killing the bacteria (Hentzer and Givskov, 2003). Various types of quorum sensing inhibitory (QSI) compounds have been derived from natural resources (Kalia, 2013), including bacteria, fungi, algae, and plant extracts. The prime QS inhibitor halogenated furanone, has been separated from the red marine algae, *Delisea pulchra* (de Nys et al., 1993; Givskov et al., 1996). Higher plants are considered as the main resource of metabolites with QSI action such as tannins from *Terminalia catappa* (Taganna et al., 2011), ajoene from garlic (Jakobsen et al., 2012), and flavonoids from *Psidium guajava* (Vasavi et al., 2014). In addition, QSI activity of synthesized compounds have been assigned such as, phenothiazines and related compounds (Varga et al., 2011), thiolactone analogs (McInnis and Blackwell, 2011), thiadiazoles derivatives (El-Gohary and Shaaban, 2013), and series of benzothiazole derivatives (Gabr et al., 2015). Also, some enzymes inactivate QS signals such as lactonase enzymes from *Bacillus* sp. (Dong et al., 2001), acylase enzymes from *Streptomyces* sp. (Park et al., 2005), and paraoxonase, a mammalian lactonase at tracheal epithelial cells, inhibit bacterial QS signals (Chun et al., 2004).

Various studies have focused on the antimicrobial activities of soil microbiota (Gang et al., 2013). *Streptomyces* are primarily distinguished by the production of antibiotics, antifungals, antivirals, antitumor, and immune-suppressants (Procópio et al., 2012). Also, *Streptomyces* secrete metabolites to compete with different microorganisms within the growing niche. Most investigations on *Streptomyces* were restricted to their antimicrobial activities; however, the antipathogenic properties of *Streptomyces* are poorly explored. QS coordinates bacterial communication and microbial pathogenicity so that QSI compounds can interfere with the QS machinery and its related virulence factors (Tang and Zhang, 2014). Compounds derived from *Streptomyces* are safe for humans and have been utilized in the treatment of pathogenic infections. Hence, screening of *Streptomyces* can deliver new QSI compounds with less ability to develop microbial resistance.

Therefore, this study was focused on screening and investigating *Streptomyces* isolated from complex microbial soil communities in Egypt for their QSI effect. Moreover, a QSI molecule was isolated and evaluated against QS regulatory genes and associated virulence factors of *P. aeruginosa*. The results provide potential targets for the construction of novel anti-pathogenic agents and permit the discovery of unique compounds that could be useful for clinical applications.

## Materials and Methods

### Screening Soil Microorganisms for Production of QS Inhibitors

#### Isolation of Soil Microorganisms

Sixteen soil samples were collected about 15 cm below the surface of the soil from different localities of Egyptian land (Oskay et al., 2004; Jeffrey, 2008) and were allowed to dry at 50°C for 10 min. One gram of the dried soil was suspended in 10 ml of sterile saline (0.9% w/v NaCl) and mixed for 20 min. Tenfold serial dilutions were prepared in the sterile saline solution with homogenous mixing. Different dilutions of soil suspension 10^7^ and 10^8^ were plotted onto ISP2 media (Williams and Cross, 1971; Jeffrey, 2008). The composition of all supplied media is provided in the **Supplementary Table S1**. The plates were incubated at 28°C for about 7–10 days. *Streptomyces* were characterized as large, glassy, rough and chalky colonies. Selected colonies were transferred from mixed culture plates to new ISP2 plates.

#### Bacterial Strains and Growth Conditions

*Chromobacterium violaceum* ATCC 12472 and CV026 reporter strains were used in the screening and the analyzing of QSI activity of the purified *Streptomyces* isolates, according to McClean et al. (1997). *P. aeruginosa* PAO1 was used as a test strain and the QS-deficient *P. aeruginosa* PAO-JP2 double mutant (Δ*lasI*::Tn10, Tcr; Δ*rhlI*::Tn501-2, Hg^r^) was included as a negative control (Pearson et al., 1997).

#### Screening of QSI Activity of the Isolated *Streptomyces*

*Streptomyces* were assessed for QS-inhibiting violacein production of the reporter strain *C. violaceum* ATCC 12472. *Streptomyces* isolates were cultivated on ISP2 plates for 6 days at 30°C. A cup of growing bacterial cells (12 mm diameter and 6 mm thickness) was placed on the surface of the bioassay plates with the upper soft LB layer inoculated with *C. violaceum* ATCC 12472 (100 μl of 1 × 10^7^ CFU/ml). The bioassay plates were incubated at 30°C for 24 h. The appearance of turbid halo pigmentless areas of CV12472 was assigned as QSI effect (McClean et al., 1997).

#### QSI Activity of S17 Isolate versus Other Isolates

According to Park et al. (2005), 50 ml ISP2-medium were inoculated with *Streptomyces* isolates S6, S12, and S17 and incubated at 30°C for 7 days. Daily samples were centrifuged at 8000 × *g* for 10 min, and then one hundred microliters of the supernatant were placed in the corresponding cup of the assay plate with a soft LB upper layer containing *C. violaceum* CV026 (100 μl of 1 × 10^7^ CFU/ml) and 50 nM of QS inducer *N*-(hexanoyl)-L-homoserine lactone. The plates were incubated at 30°C for 24 h with monitoring of the violet color. The diameter of pigmentless turbid halo areas of CV026 around the cup was measured.

### Nature of QSI Compounds

In order to estimate the nature of QS-inactivating molecules, the cell-free suspension of isolate S17 was inactivated either by heat or by the treatment with proteinase K. The cell-free supernatant was heated at 95°C for 15 min. The supernatant (100 μl) was also incubated with proteinase K (5 mg) for 1 h at 55°C. Treated suspensions then tested for inhibition of violacein production with CV026 compared to the untreated culture supernatant (100 μl) as a positive control. The ethyl acetate extract (EtOAc) of the cell-free supernatant (100 μl; 1 mg/ml) was also compared to the untreated culture supernatant as a positive control and the solvent as a negative control (Musthafa et al., 2011).

### Chromatographic Investigation of the Ethyl Acetate Extract of S17 Isolate

For column chromatography, silica gel G60-230 (Merck, Germany) and Sephadex LH-20 (Sigma–Aldrich, USA) were used. Analytical thin layer chromatography (TLC) was performed on a pre-coated silica gel 60 GF_254_ (Merck or Machery-Nagel, Germany). The 1D and 2D NMR spectra were performed on Bruker-400 Ascend^TM^ spectrometer using CDCl_3_ or dimethyl sulfoxide deuterated (DMSO-*d*_6_) as solvents. The electrospray mass spectrometry (ESMS) experiments were conducted with the 3200 Q-trap LC/MS/MS system (Applied Biosystems, Foster City, CA, USA) Analyst version 1.4.1 software (MDS Sciex; Toronto, CA, USA).

The TLC chromatogram of the obtained EtOAc extract [CH_2_Cl_2_–MeOH (95: 5 v/v)] revealed the presence of three major spots on visualization with 10% H_2_SO_4_ spray reagent and heating at 110°C for 1 min. The first spot (*R*_f_ 0.65) had no response both under UV_254_ and under UV_366_ lights; on visualization, however, it gave a pale orange color. The other two spots (*R*_f_ 0.25 and 0.39) quenched UV_254_ light and gave a brown color.

The EtOAc extract (700 mg) was applied to a silica gel chromatographic column (35 g), packed in CH_2_Cl_2_ 100% and eluted with CH_2_Cl_2_–MeOH mixtures with different polarities to afford compounds **1** (*R*_f_ 0.65, 18 mg), **2** (*R*_f_ 0.39, 7 mg), and **3** (*R*_f_ 0.25, 10 mg). A detailed isolation procedure is presented in Supplementary Materials.

### Molecular Identification of *Streptomyces* Isolate S17

The DNA of *Streptomyces* isolate S17 was extracted according to Nikodinovic et al. (2003). A partial fragment of 16S rRNA gene (V1–V6) was amplified and sequenced using 16S rRNA primers (**Table 1**; ABI 3730xl sequencer, Applied Biosystems). Sequencing analysis was performed using the BLAST search tool of the National Center for Biotechnology Information (NCBI). Nucleotide similarity was verified through the sequence-matching tool of the Ribosomal Database Project (Maidak et al., 2000).

**Table 1:** PCR primers utilized in 16S rRNA sequencing and in RT-PCR.

Gene name	Type	Primer sequence	Annealing temperature (°C)	Amplicon size (bp)
16S rRNAV1-V6	Fw	5′**–**AGAGTTTGATCMTGGCTCAG**–**3′	46	989
	Rev	5′**–**ACGAGCTGACGACARCCATG**–**3′		
*ropD* PA0576	Fw	5′**–**CGAACTGCTTGCCGACTT**–**3′	56	131
	Rev	5′**–**GCGAGAGCCTCAAGGATAC**–**3′		
*lasI* PA1432	Fw	5′**–**CGCACATCTGGGAACTCA**–**3′	56	176
	Rev	5′**–**CGGCACGGATCATCATCT**–**3′		
*lasR* PA1430	Fw	5′**–**CTGTGGATGCTCAAGGACTAC**–**3′	55	133
	Rev	5′**–**AACTGGTCTTGCCGATGG**–**3′		
*lasA* 1871	Fw	5′**–** CGCTGAATGACGACCTGTT**–**3′	56	143
	Rev	5′**–** CTTTCGGGTTGATGCTGTAGT**–**3′		
*lasB* PA3724	Fw	5′**–**GGTAGAACGCACGGTTGT**–**3′	55	165
	Rev	5′**–**GGCAAGAACGACTTCCTGAT**–**3′		
*rhlI* PA3476	Fw	5′**–**GTAGCGGGTTTGCGGATG**–**3′	58	101
	Rev	5′**–**CGGCATCAGGTCTTCATCG**–**3′		
*rhlR* PA3477	Fw	5′**–**GCCAGCGTCTTGTTCGG**–**3′	58	160
	Rev	5′**–**CGGTCTGCCTGAGCCATC**–**3′		
*pqsA* PA0996	Fw	5′–GACCGGCTGTATTCGATTC–3′	55	74
	Rev	5′–GCTGAACCAGGGAAAGAAC–3′		
*pqsR* PA0964	Fw	5′–CTGATCTGCCGGTAATTGG–3′	55	142
	Rev	5′–ATCGACGAGGAACTGAAGA–3′		


Phylogenetic analysis was accomplished utilizing CLUSTAL W software (Thompson et al., 1994). The evolutionary trees were inferred from the neighbor-joining method by utilization of MEGA version V (Saitou and Nei, 1987; Kumar et al., 2001) using default parameters. The stability of the relationships was assessed by performing bootstrap analyses of neighbor-joining data based on 1,000 resampling.

### Influence of the Isolated Compounds on Virulence Factors of *P. aeruginosa* PAO1

#### Total Protease Production

The overnight cultures of *P. aeruginosa* PAO1 (0.5 ml) were propagated in 5 ml LB broth containing 1 mg/ml of each purified compound (docosanoic acid, borrelidin, and 1*H*-pyrrole-2-carboxylic acid) at 37°C for 18 h with shaking at 150 rpm. The cell-free supernatants of treated and untreated PAO1 were collected. PAO-JP2 was propagated under the same conditions as a negative control. The supernatant of *P. aeruginosa* (700 μl) was mixed with an equal volume of skimmed milk 1.25% w/v and kept at 37°C for 15 min and OD_600_ was measured. The total protease activity was quantified and compared to untreated *Pseudomonas* PAO1 in triplicates according to the modified skim milk method (El-Mowafy et al., 2014b).

#### Elastase Activity

Elastolytic activity was assessed both in the presence and in the absence of 1 mg/ml of each purified compound according to the elastin Congo red assay method (Musthafa et al., 2011). The cell-free supernatant of *P. aeruginosa* PAO1 was mixed with an equal volume of Elastin Congo red (10 mg) in 100 mM Tris/HCl (pH 7.5) for 4 h at 37°C. The mixture was centrifuged at 8000 × *g* for 10 min to remove the insoluble Congo red pigment. Elastase activity of the treated supernatants was measured at OD_495_, compared to the untreated culture of PAO1.

#### Effect on Pyocyanin Production

The pure compounds (1 mg/ml) were added separately to King’s A media. The media were inoculated with *P. aeruginosa* PAO1 and incubated at 37°C for 48 h while shaking at 150 rpm. Pyocyanin pigment was extracted by being mixed with chloroform (3 ml). After centrifugation (8000 × *g* for 10 min), the lower organic layer was withdrawn and mixed with 1 ml of 0.2 N HCl to elaborate the acidic red pyocyanin. Pyocyanin level was calculated by measuring the absorbance of the aqueous red phase at OD_520_ (Essar et al., 1990). The pyocyanin level of the double mutant (negative control) and untreated PAO1 (positive control) were also quantified. The assay was performed in triplicates.

#### Effect of the Pure Compounds on the Growth of *P. aeruginosa* PAO1

*Pseudomonas aeruginosa* PAO1 was propagated in the presence of 1mg/ml of each purified compound docosanoic acid, borrelidin and 1*H*-pyrrole-2-carboxylic acid. Control untreated PAO1 was cultivated under the same conditions. Samples of each reaction were taken every hour and OD_600_ was measured.

Moreover, the viable count of PAO1 treated with 1 mg/ml of docosanoic acid, borrelidin, and 1*H*-pyrrole-2-carboxylic acid was estimated using the pour plate assay technique (Standards Australia, 1995). Ten fold serial dilutions of bacterial samples were prepared. A sample (1 ml) of each mixture was collected and the viable *Pseudomonas* colonies were counted over 18 h and compared to the untreated PAO1.

#### Effect on the Expression Level of QS Genes

*Pseudomonas aeruginosa* PAO1 was cultivated in the presence of 1*H*-pyrrole-2-carboxylic acid (1 mg/ml) until the middle of the exponential growth phase (OD_600_; 0.4–0.5). The untreated PAO1 and PAO-JP2 were similarly propagated as positive and negative controls, respectively. The total RNA was extracted using TRIzol reagent according to the manufacturer’s instructions. DNA was removed using the gDNA wipeout buffer, and the complementary DNA was synthesized using the QuantiTect Reverse Transcription kit (QIAGEN, Hilden, Germany). Quantitative PCR was used to measure the effect of 1*H*-pyrrole-2-carboxylic acid on the expression of QS genes *lasI, lasR, lasA, lasB, rhlIR, rhlI, pqsA* and *pqsR* in treated and untreated PAO1 cultures in duplicates. Amplification and expression were performed using FIREPol EvaGreen and qPCR Mix (Solis BioDyne, Tartu, Estonia) using primers (**Table 1**). The expression of the quantified genes was normalized to the expression of the housekeeping gene *ropD*. The level of gene expression of treated PAO1 was calculated relative to that in the untreated PAO1.

### Factors Affecting QSI Activity of S17 Isolate

Different media were tested for their effect on QS-quenching activity of S17 isolate including; the GSS medium, the GSM medium, the M2 medium and the ISP2 medium (media composition **Supplementary Table S1**). Each medium was assessed in triplicate (Zhu et al., 2007).

The influence of various carbon sources on QSI effect of S17 was also studied using the ISP2 medium containing 0.4% w/v glucose and the ISP2 medium in which glucose was substituted by an equal weight of lactose, sucrose, and starch (Pandey et al., 2005).

The influence of medium pH and incubation temperatures on the QSI action of the S17 isolate was also estimated. The ISP2 media with initial pH values of 5, 6, 7, and 8 were inoculated and grown at 30°C on a rotary shaker (Shel lab, USA) at 150 rpm for 7 days. Also, the ISP2 medium was inoculated and incubated at 25, 30, and 37^o^C for 7 days while shaking at 150 rpm (da Silva et al., 2012). Aliquots of 100 μl (1 mg/ml) from various conditions were assessed for QSI activity (da Silva et al., 2012).

### Statistical Analysis

Mean and standard deviation were calculated for QSI activity using the GraphPad Instate software package (version 3.05). Statistical analysis was accomplished with the Tukey–Kramer multiple-comparison method where a statistically significant *p*-value <0.05 or *p* < 0.01.

## Results

### Bacterial Isolation and Purification

A total of 65 different isolates of *Streptomyces* were purified from 16 soil samples collected from different localities of Egyptian soil including Dakahlia, Damietta, Cairo and Suez governorates. The types of the collected soil samples were specified in the **Supplementary Table S2**. The *Streptomyces* isolates were characterized by tough, leathery, pigmented colonies and filamentous growth. Also, they had a branched network of mycelia with conidiophores at the terminal end of aerial mycelia.

### QS Inhibiting Activity of *Streptomyces* Isolates

**FIGURE 1:**
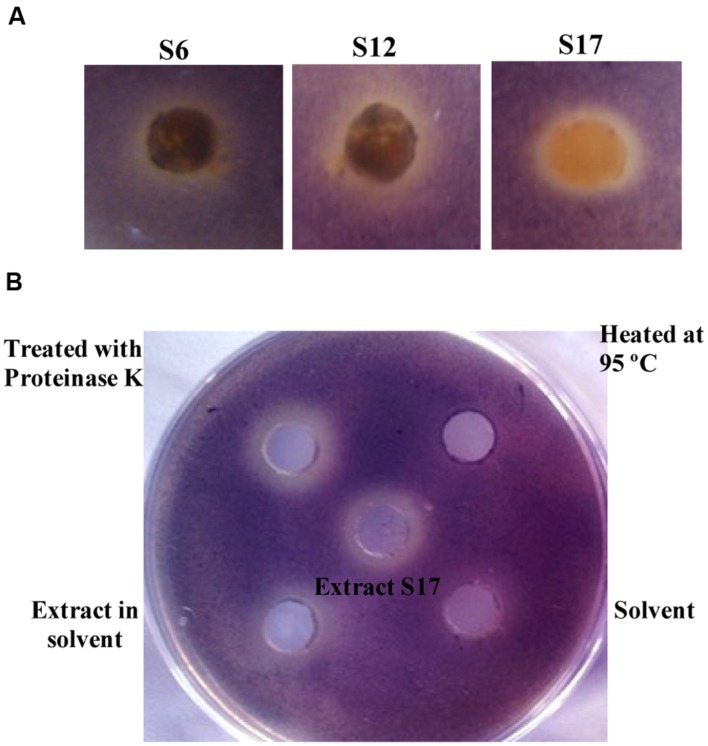
**Screening of quorum sensing (QS) inhibiting activity of tested *Streptomyces* isolates and the nature of QS inhibitory components of *Streptomyces* S17.** **(A)** *Chromobacterium violaceum* ATCC 12472 culture (100 μl of 1 × 10^7^ CFU/ml) was inoculated into 5 ml LB soft agar (0.5% agar) and overlaid on the surface of LB agar plate. A cup of growing *Streptomyces* isolates was placed into the surface of the bioassay plate and the plates were incubated at 30°C for 24 h for disappearance of violet color of *C. violaceum*. Isolates S6, S12, and S17 showed QS inhibiting activity. **(B)** *Chromobacterium violaceum* CV026 (100 μl of 1 × 10^7^ CFU/ml) was inoculated into 5 ml LB soft agar (0.5% agar) containing 50 nM *N*-(hexanoyl)-L-homoserine lactone, and the mixture was overlaid on the surface of LB agar plate. The plates were incubated at 30°C for 24 h and QSI activity was assigned by disappearance for the violet color. The cell free supernatant of S17 (1 mg/ml) treated with 5 mg proteinase K maintained QSI activity. The ethyl acetate extract of S17 (1 mg/ml) and solvent control were applied into wells at opposite sides of a Petri dish containing a layer of water agar. The extract in ethyl acetate also retained the QSI activity, compared to solvent control.

All purified *Streptomyces* isolates were screened for their QSI effect using *C. violaceum* ATCC 12472 (**Figure 1A**) among which, the three isolates: S6, S12, and S17 inhibited violet pigment formation of *C. violaceum* ATCC 12472 without affecting bacterial growth. The prepared extracts of the three isolates, S6, S12, and S17, were assessed for their QSI activity using the CV026 reporter strain (**Supplementary Figure S1**) (McClean et al., 1997). Isolate S17; purified from the soil sample collected from Mansoura University Gardens, Dakahlia, Egypt; revealed the maximum QSI activity after 4 and 5 days of cultivation. Isolates S6 and S12 showed lower QSI action than S17 isolate at the 5th day of cultivation (**Supplementary Figure S1**). Consequently, isolate S17 was further studied.

### Nature of AHL-inactivating Compound

The supernatant of *Streptomyces* S17 mixed with proteinase enzyme retained its original QSI level. However, heating the supernatant up to 95°C caused a marked loss of QSI effect (**Figure 1B**). The QSI activities of the cell-free supernatant, the ethyl acetate extract, the proteinase K-treated extract and the heat-treated extract from S17 isolate were determined and compared to the control (**Supplementary Figure S1**).

### Spectral Analysis of the Isolated Metabolites from *Streptomyces* S17

**FIGURE 2:**
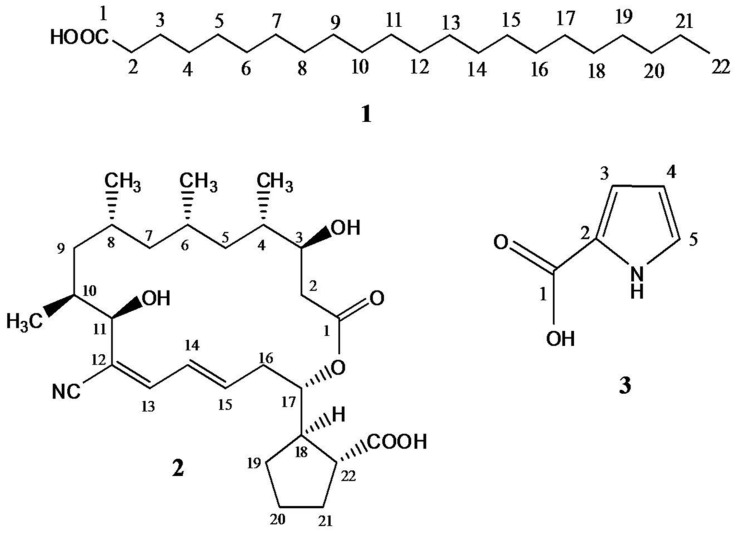
**Structures of the isolated metabolites (**1–3**); behenic acid (docosanoic acid), borrelidin, and 1*H*-pyrrole-2-carboxylic acid from *Streptomyces* S17**.

Chromatographic investigation of the bioactive EtOAc extract of *Streptomyces* S17 had detected three compounds: **1**, **2**, and **3**. Compound **1**, behenic acid (docosanoic acid; **Figure 2**), was obtained as colorless waxy semisolid; its ^1^H NMR spectrum (CDCl_3_, 400 MHz) showed proton signals at δ_H_ 10.37 (1H-1), 1.41 (2H, H-2), 1.63 (2H, H-3), 1.22 (20H, H-4:19), 0.89 (4H-20:21), and 0.87 (3H, H-22). ESMS^-^ peaks at *m/z* 339.3 [M–H], 325.3 [M–CH_3_], 311.3 [M–C_2_H_5_], 297.3 [M–C_3_H_7_], 283.4 [M–C_4_H_9_], 269.3 [M–C_5_H_11_], 255.2 [M–C_6_H_13_, base peak], and 241.3 [M–C_7_H_15_] (**Supplementary Figures S2**, **S3**, and **S15**).

Spectral ^1^H NMR data of compound **2** (borrelidin) (**Figure 2**) is presented in **Table 2.** Spectral ^1^H NMR analysis of compound **2** indicated the presence of three olefinic protons resonating at δ_H_ 6.13–6.74 (C_13-15_), three down field proton doublets at δ_H_ 3.79–4.89 (C_3,11,17_) and a range of aliphatic resonances at δ_H_ 0.73–2.62. Furthermore, the ^13^C NMR data of compound **2** (**Table 2**) showed 28 carbon signals. The key signals included three hydroxyl methine carbons at δ_HC_ 70.0 (C_3_), 73.2 (C_11_) and 76.3 (C_17_), a conjugated nitrile group at δ_C_ 116.0 (CN) and two carbonyl groups at δ_H_ 179.9 (–COOH) and 172.9 (–OCO) (**Supplementary Figures S4**–**S11**).

**Table 2:** NMR data of compound 2 isolated from *Streptomyces* S17, CDCl_3_ (400 MHz for ^1^H and 100 MHz for ^13^C NMR) and *J* (Hz).

Position	δ_C_ (ppm)	DPT135	δ_H_ (ppm), *J* (Hz)	HMBC
1	172.2	C	–	
2	39.1	CH_2_	*H*_a_: 2.31, m*H*_b_: 2.38, m	70.0, 172.2
3	70.0	CH	3.79, d (8)	
4	35.2	CH	1.82, m	
5	43.0	CH_2_	*H*_a_: 0.80, m*H*_b_: 1.20, m	70.3
6	27.2	CH	1.54, m	
7	47.8	CH_2_	*H*_a_: 0.88, m*H*_b_: 1.03, m	
8	26.3	CH	1.60, m	
9	37.5	CH_2_	*H*_a_: 0.68, m*H*_b_: 0.95, m	
10	35.6	CH	1.54, m	
11	73.2	CH	4.03, d (8)	144.0, 116.0, 14.9, 35.6
12	118.1	C	–	
13	144.0	CH	6.74, d (12.0)	73.2, 116.0, 138.4
14	127.0	CH	6.29, dd (12, 16)	
15	138.4	CH	6.08, ddd (12, 8, 4)	
16	35.9	CH_2_	2.48, 2H, m	
17	76.3	CH	4.89, d (12)	172.2
18	45.8	CH	2.62, pentet (8)	
19	29.7	CH_2_	*H*_a_: 1.28, m*H*_b_: 1.84, m	
20	25.2	CH_2_	1.75, 2H, m	
21	31.2	CH_2_	*H*_a_: 1.75, m*H*_b_: 1.91, m	
22	48.3	CH	2.48, q (8)	
4 (CH_3_)	17.0	CH_3_	0.77, d (8)	43.0, 35.2
6 (CH_3_)	18.2	CH_3_	0.74, d (4)	47.8, 43.0, 27.2
8 (CH_3_)	20.1	CH_3_	0.77, d (8)	47.8, 37.5, 26.3
10 (CH_3_)	14.9	CH_3_	0.98, d (4)	73.2, 35.6
CN	116.0	C	–	
COOH	179.9	C	–	


Compound **3** (1*H*-pyrrole-2-carboxylic acid; **Figure 2**) was obtained as a light brown solid; it quenched UV_254_ light and gave a grayish brown color upon spraying it with 10% H_2_SO_4_. It has *R*_f_ value 0.25 using CH_2_Cl_2_–MeOH (95:5 v/v). Its molecular formula was established to be C_5_H_5_NO_2_ as deduced from ESMS^-^ at *m/z* 110.0 [M–H]. ^1^H-NMR spectrum (DMSO-*d*_6_, 400 MHz) showed proton signals at δ_H_ 12.19 (1H, br s, –COOH), 11.70 (1H, s, –NH), 6.96 (1H, br s, H-5), 6.73 (1H, d, *J* = 1.2 Hz, H-3) and 6.13 (1H, dd, *J* = 1.96, 1.2 Hz, H-4). APT experiment (DMSO-*d*_6_, 100 MHz) showed two quaternary carbon signal at δ_C_ 162.3 (C-1) and 123.4 (C-2), and three methine carbon signal at δ_C_ 123.8 (C-5), 115.1 (C-3) and 109.7 (C-4) (**Supplementary Figures S12**, **S13**, **S14** and **S16**).

### Inhibition of Virulence Factors of PAO1

**FIGURE 3:**
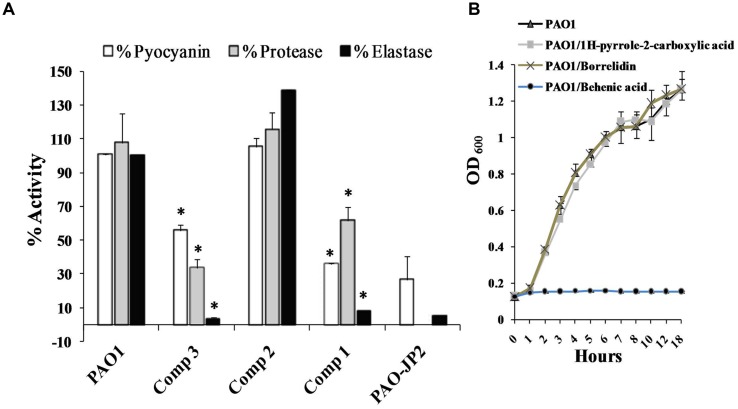
**Effect of the purified compounds behenic acid, borrelidin and 1*H*-pyrrole-2-carboxylic acid (**1–3**) on virulence factors of PAO1.** **(A)** *Pseudomonas aeruginosa* PAO1 treated with 1 mg/ml of pure compounds was assessed for elastase, protease and pyocyanin compared to untreated PAO1 as positive control and PAO-JP2 as negative control. Compounds (**1** and **3**) caused a significant elimination of elastase, protease, and pyocyanin compared to control untreated PAO1 **(B)** *P. aeruginosa* PAO1 was propagated with 1 mg/ml behenic acid or borrelidin or 1*H*-pyrrole-2-carboxylic acid, 1*H*-pyrrole-2-carboxylic acid, and borrelidin did not affect bacterial growth, however, behenic acid caused a marked decrease in the growth of PAO1 compared to the control untreated cultures (highly significant with ^∗^*p* < 0.01).

Treating *P. aeruginosa* PAO1 with 1 mg/ml of either docosanoic acid or 1*H*-pyrrole-2-carboxylic acid caused a significant decrease in *Pseudomonas* virulence factors (**Figure 3A**). 1*H*-pyrrole-2-carboxylic acid significantly reduced pyocyanin, protease, and elastase by 44, 74, and 96% (*p* < 0.01). On the same instance, docosanoic acid decreased total pyocyanin, protease, and elastase formation by 64.45, 46.1, and 91.8% with *p* < 0.01.

### Effect of the Purified Compounds on Bacterial Viability

Determination of *Pseudomonas* viability in the presence of 1 mg/ml of docosanoic acid, or borrelidin or 1*H*-pyrrole-2-carboxylic acid is important to estimate whether their effects were caused by the inhibition of QS or as a result of bacteriostatic/bactericidal effects. PAO1 cultured with 1 mg/ml of 1*H*-pyrrole-2-carboxylic acid had the same bacterial count (1.64 × 10^8^ CFU/ml) as that of untreated PAO1 (1.61 × 10^8^ CFU/ml). However, the docosanoic acid (1 mg/ml) caused a marked decrease in the bacterial growth of the treated culture (2 × 10^6^ CFU/ml) compared to the untreated PAO1 (1.61 × 10^8^ CFU/ml). Also, the OD_600_ of *P. aeruginosa* PAO1 propagated with 1*H*-pyrrole-2-carboxylic acid or borrelidin (1 mg/ml) showed the same growth curve of control untreated PAO1. However, 1 mg/ml of docosanoic acid revealed a marked weak growth compared to untreated PAO1 cultures (**Figure 3B**).

### Elimination of QS-Cascade of *P. aeruginosa* PAO1

**FIGURE 4:**
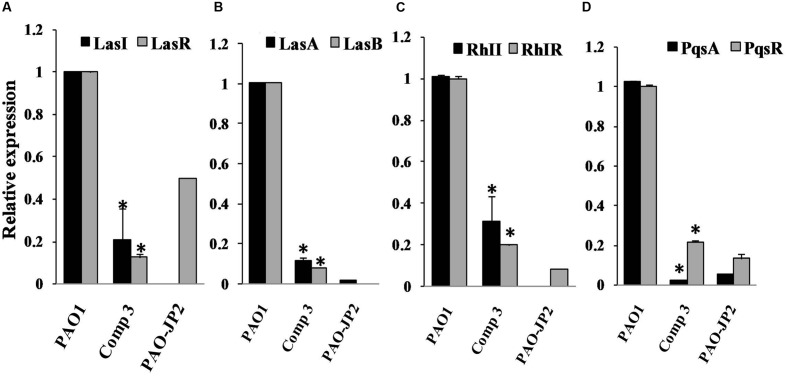
**The influence of compound **3** (1*H*-pyrrole-2-carboxylic acid) on QS regulatory circuits of *P. aeruginosa* PAO1; Real-time PCR was used to measure the effect of 1*H*-pyrrole-2-carboxylic acid (1mg/ml) on the expression of QS genes. 1*H*-pyrrole-2-carboxylic acid repressed QS genes of *P. aeruginosa* PAO1 **(A)***lasI* and *lasR*, **(B)***lasA* and *lasB*, **(C)***rhlI* and *rhlR* and **(D)***pqsA* and *pqsR* compared to untreated PAO1 (positive control) and double mutant PAO-JP2 (negative control).** The expression of each gene was normalized to the expression of reference gene *ropD*. The level of gene expression of PAO1 with 1*H*-pyrrole-2-carboxylic acid was calculated relative to that of the untreated PAO1 (highly significant with ^∗^*p* < 0.01).

Relative expressions of QS cascade *lasI*, *lasR*, *lasA*, *lasB*, *rhlI*, *rhlR*, *pqsA*, and *pqsR*, were assessed for PAO1 treated with 1*H*-pyrrole-2-carboxylic acid and untreated cultures. The standard curve of the reference gene *ropD* and QS genes showed *R*^2^ values 0.99–0.96. Moreover, melting reports indicated the formation of pure amplicons. 1*H*-pyrrole-2-carboxylic acid significantly eliminated the expression of *las* genes including *lasI, lasR, lasA*, and *lasB*, by 80, 87, 88, and 92%, respectively, with *p* < 0.01 (**Figure 4**). 1*H*-pyrrole-2-carboxylic acid also significantly inhibited *rhl*/*pqs* cascades involving *rhlI, rhlR, pqsA*, and *pqsR* genes by 69, 89, 97, and 78%, respectively (*p* < 0.01, **Figure 4**).

### 16S rRNA Sequencing

**FIGURE 5:**
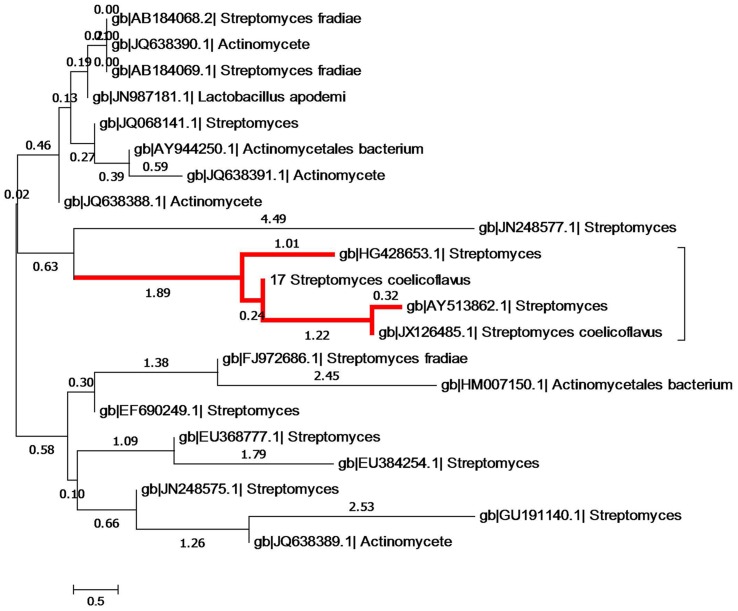
**Phylogenetic tree showing evolutionary relationship of S17 with other members of *Streptomyces* on the basis of 16S rRNA sequences evolutionary distances.** Nucleotide alignment and phylogenetic analysis was performed using CLUSTAL W. The nucleotide similarity values were calculated and the evolutionary trees were detected from the neighbor joining method using MEGA version V. The red high light indicates the phylogeneitc position of isolate S17, it clustered with *Streptomyces coelicoflavus* with high sequences similarity (100%).

The 16S rRNA gene (989 bp) of isolate S17 was sequenced and had been submitted to GenBank (NCBI) under accession number KJ855087. In addition, the generated 16S sequence of the S17 isolate was successfully identified within genus *Streptomyces*, according to the neighbor-joining tree (**Figure 5**). Alignment of the generated 16S rRNA sequence retrieved that S17 was pairwise aligned with *Streptomyces coelicoflavus* with sequence similarity and identity of 100%.

### The Impact of Media, pH, and Temperature changes on QSI Activity of S17 Isolate

**FIGURE 6:**
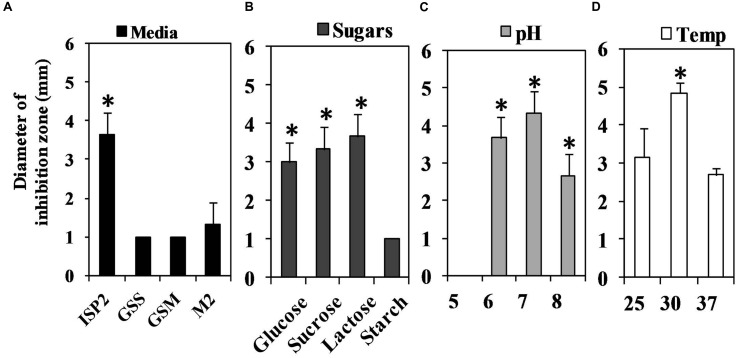
**Assessment of the QS inhibitory activity of *Streptomyces* isolate S17 under different cultivation conditions using *Chromobacterium violaceum* CV026 bioassay technique at the 5th day of incubation.** **(A)** Different media GSS, GSM, M2 and ISP2 media were tested for QSI potential and the highest yield was obtained using ISP2 medium. **(B)** ISP2 medium supplied with glucose or lactose or sucrose (0.4% w/v) revealed high QSI yield compared to medium contained starch (0.4% w/v). **(C)** The effect pH (5–8) of ISP2 media on QSI action of S17 was also performed and media with pH 6–7 showed optimum QSI effect. **(D)** Cultivation of S17 at 30°C associated with a significant QSI yield. (significant with ^∗^*p* < 0.05).

Different media of diverse compositions produced low QSI activity compared to the ISP2 medium (**Figure 6A**). The maximum QSI activity of S17 was attained in the presence of glucose 0.4% w/v at the 4th and 5th days of the growth. Sucrose and lactose-supplemented media also produced almost the same QSI yield of the glucose supplemented medium but with a delayed activity. The highest QSI levels using sucrose and lactose supplements were obtained at the 5th day of incubation. However, starch produced low QSI effect (**Figure 6B**).

The influence of different pH values on QSI action of S17 was determined (**Figure 6C**). The highest yield was obtained at pH 7 during the 4th and 5th days of incubation while the QSI activity at pH 5 was significantly reduced. The optimum temperature for QSI action was attained at 30°C as shown in **Figure 6D.**

## Discussion

Quorum sensing regulates cell density and virulence factors such as biofilm formation, metabolites production and host-microbe interaction (Hentzer and Givskov, 2003). Thus, interference with QS will provide a mean for treating the chronic bacterial infection. Various QSI compounds have been identified from either natural resources (Zaki et al., 2013) or chemical compounds (El-Mowafy et al., 2014a). For decades, *Streptomyces* have been considered an important source of antibiotics and other metabolites. With the development of antimicrobial resistance, attention has been directed towards the exploration of antipathogenic agents. Such compounds inhibit the signaling and the virulence of bacterial pathogens (Persson et al., 2005).

In this study, *Streptomyces* isolates were purified from soil samples and characterized as round, chalky colonies, with different colors (white, greenish brown, gray, pink, or other colors) (Williams and Cross, 1971). McClean et al. (1997) research group construct *C. violaceum* CV026 as a violacein negative double mutant which has been used for assessing QSI activity of chemically synthesized compounds such as furanones and other natural products (Martinelli et al., 2004). AntiQS molecules inhibit pigment formation of *C. violaceum* ATCC 12472 without affecting the growth of the reporter strain. In this study, three isolates: S6, S12, and S17, inhibited the violet pigment formation of *C. violaceum* ATCC 12472 without affecting bacterial growth (**Figure 1A**). Previous studies have identified QSI activity of bacteria isolated from soil, such as *Proteobacteria* purified from China (Weng et al., 2012) and *Arthrobacter* identified from Malaysian soil (Chong et al., 2012).

The nature of QS inhibitors may be enzymatic or non-enzymatic (Du et al., 2014). Mechanistically, the enzymatic inhibitors of QS signals degrade either the lactone or acyl-chains of the AHLs (Dong and Zhang, 2005; Park et al., 2005). In this study, incubation with proteinase K did not affect the QSI activity of S17 (**Figure 1B**) compared to the untreated control. Furthermore, the organic extract of *Streptomyces* S17 retained quorum quenching activity which indicated the non-enzymatic nature of the QSI metabolites produced by S17. However, QSI action of S17 was lost by heating up to 95°C, which may be attributed to the heat instability of the QSI components of the extract. Similarly, previous studies reported the isolation and the characterization of QSI compounds from *Streptomyces* isolates such as cinnamic acid and dipeptide proline–glycine from a marine invertebrate *Streptomyces* (Naik et al., 2013). Also, *Streptomyces* TOHO-O348 and TOHO-Y209 produce piericidins with a marked QSI effect on *C. violaceum* CV026 (Ooka et al., 2013).

Chromatographic investigation of the bioactive EtOAc extract of *Streptomyces* S17 detected three major compounds **1**, **2,** and **3**. The spectral data of compound **1** indicated a typical pattern of a long chain saturated fatty acid, with the molecular formula, C_22_H_44_O_2_, as deduced from ESMS^-^. Thus, compound **1** was identified as behenic acid (docosanoic acid; **Figure 2**). It is noted that it is the first report of isolation of behenic acid from *Streptomyces* sp. A comparison of spectral NMR data of compound **2** (**Table 2**) with literature, assumed that compound **2** is the previously described macrolide, borrelidin (**Figure 2**) (Kuo et al., 1989; Yassien et al., 2015). This assumption was confirmed using heteronuclear multiple bond correlation (HMBC) experiment. Although, borrelidin (treponemycin) is isolated before from *Streptomyces* sp., this is the first purification and characterization of this compound from *S. coelicoflavus*. Compound **3** was identified as 1*H*-pyrrole-2-carboxylic acid (**Figure 2**), which has been detected in *Streptomyces* sp. (Corpe, 1963; Zhang et al., 2011), however, this is the first isolation of this compound from *S. coelicoflavus*.

The purified compounds, 1*H*-pyrrole-2-carboxylic acid and behenic acid, posed QSI activity against *P. aeruginosa* PAO1 with a significant reduction in elastase, total protease, and pyocyanin (**Figure 3**). Behenic acid (1 mg/ml) reduced *Pseudomonas* viability. Hence, its effect on *Pseudomonas* virulence factors was attributed to the inhibition of bacterial growth (**Figure 3B**). Fatty acids of various chain lengths are known for their antimicrobial effects. Non-dissociated fatty acids dissolve phospholipids in the cytoplasmic membrane and disrupt bacterial viability (Kabara et al., 1972). On the other hand, 1*H*-pyrrole-2-carboxylic acid, isolated from S17, is a heterocyclic pyrrole derivative. It exhibited its QSI activities by eliminating the QS cascades *las*, *rhl*, and *pqs* (**Figure 4**). 1*H*-pyrrole-2-carboxylic acid caused a significant decrease in QS-controlled virulence factors without affecting bacterial viability (**Figure 3B**). Likewise, the red algae, *D. pulchra*, produce a class of halogenated furanones known as fimbrolides (de Nys et al., 1993). They competitively inhibit and interrupt the signaling cascade of *Vibrio* sp. and *Escherichia coli* (Kjelleberg et al., 1997). Also, dihydropyrrolones derivatives of fimbrolides inhibit AHL-mediated QS, bacterial adhesion and prevent biofilm assembly in several pathogenic organisms without affecting bacterial viability (Baveja et al., 2004; Ho et al., 2010).

For further investigation, QSI activity of isolate S17 was evaluated under different culture conditions. ISP2 medium showed the highest QSI effect as it was supplied with 0.4% w/v glucose as the main carbon source. However; other media such as GSS and GSM contained starch as a carbon source that is poorly utilized by *Streptomyces* (**Figure 6A**). On the other hand, high glucose content up to 2% w/v in GSS and GSM and 1% w/v in the M2 medium, had an inhibitory effect on the QSI potential of isolate S17 (**Figure 6B**). In a similar manner, the biosynthesis of avilamycin from *S. viridochromogenes* AS4.126 is repressed by elevated glucose concentrations (Zhu et al., 2007). Moreover, propagation of S17 at pH 6–7 produced the highest QSI outcome (**Figure 6C**). Also, cultivation of S17 at 30°C revealed a significant QSI yield (**Figure 6D**). Likewise, the optimum clavulanic acid production from *Streptomyces* DAUFPE 3060 is attained by propagation at 32°C and at pH values 6 or 7 (Viana et al., 2010).

## Conclusion

The inquiries and findings of this study are critical for the assessment of the QSI activity of *Streptomyces* sp. isolated from Egyptian soil. This research explored the QSI effect of *Streptomyces* S17 obtained from Egyptian soil with a 100 % similarity to *S. coelicoflavus*. This is the first study assigned purification of behenic acid (docosanoic acid), borrelidin and 1*H*-pyrrole-2-carboxylic acid from *S. coelicoflavus*. The major metabolite, 1*H*-pyrrole-2-carboxylic acid, eliminated the expression of QS cascade and the pathogenic factors of *P. aeruginosa* PAO1 on both phenotypic and genotypic levels. Such small molecule provides a useful scaffold for synthesis and construction of novel anti-virulence drugs derived from natural sources. This could potentially lead to the development of new QS inhibitors with therapeutic applications. Furthermore, it opens the way for screening other soil microbiota for QS inhibitors. Still, applications require additional toxicological studies to declare *in vivo* activity.

## Author Contributions

All authors listed, have made substantial, direct and intellectual contribution to the work, and approved it for publication. MIS and AME-M purified *Streptomyces* from soil samples. RH, MIS, AME-M, and SS studied QSI effects of the extracts and purified compounds. FMA: performed extraction, isolation and spectroscopic analyses of the isolated compounds.

## Conflict of Interest Statement

The authors declare that the research was conducted in the absence of any commercial or financial relationships that could be construed as a potential conflict of interest.
