# Supplementary material for: Quorum Sensing Inhibiting Activity of Streptomyces coelicoflavus Isolated from Soil
Source: Front Microbiol. 2016 May 13;7:659. doi: 10.3389/fmicb.2016.00659 (PMC4866617; doi:10.3389/fmicb.2016.00659)
Supplement: Supplementary file 1 [file Data_Sheet_1.DOCX]

Supplementary Material

**Quorum sensing inhibiting activity of *Streptomyces coelicoflavus* isolated from soil**

**Ramadan Hassan^1^, Mona Shaaban ^1*^, Fatma M. Abdel Bar ^2^, Areej El-Mahdy^1^, Shady Shokralla^1, 3^**

^1^ Microbiology Department, Faculty of Pharmacy, Mansoura University, Mansoura, Egypt.

^2^ Pharmacognosy Department, Faculty of Pharmacy, Mansoura University, Mansoura, Egypt.

^3^ Biodiversity Institute of Ontario, Department of Integrative Biology, University of Guelph, Guelph, Ontario, Canada.

***Corresponding author:**

Mona Shaaban, Faculty of Pharmacy, Mansoura University, Egypt

E-mail: mona_ibrahem@mans.edu.eg

**Keywords**: Quorum sensing inhibitor, soil *Streptomyces*, *Streptomyces coelicoflavus*, *Pseudomonas* virulence factors, 1*H*-pyrrole-2-carboxylic acid, borrelidin, behenic acid, antipathogenic.

| **Contents** | **Page** |
| --- | --- |
| Chromatographic investigation of the EtOAc extract of S17 isolate | **3** |
| **Table S1.** Composition of the supplied media | **4** |
| **Table S2.** Distribution of *Streptomyces* isolates purified from different localities. | **5** |
| **Figure S1**. A) Assay of quorum sensing inhibiting activity of the tested *Streptomyces* isolates using *C. violaceum* CV026, isolates S17 and S12 showed early QSI effects but S6 showed lower yield with delayed production B) Quantitative bioactivity of the cell free supernatant, ethyl acetate extract from S17, proteinase K treated extract, heated extract compared to solvent control (**; *p* <0.001). | **6** |
| **Figure S2:** ^1^HNMR spectrum (CDCl_3_, 400 MHz) of compound **1** behenic acid (docosanoic acid). | **7** |
| **Figure S3:** APT spectrum (CDCl_3_, 100 MHz) of compound **1** behenic acid (docosanoic acid). | **7** |
| **Figure S4:** ^1^H NMR spectrum (CDCl_3_, 400 MHz) of compound **2** (borrelidin). | **8** |
| **Figure S5:** Expanded ^1^H NMR spectrum, from 0.6-2.0 ppm (CDCl_3_, 400 MHz) of compound **2** (borrelidin). | **8** |
| **Figure S6:** Expanded ^1^H NMR spectrum, from 2.0-5.1 ppm (CDCl_3_, 400 MHz) of compound **2**(borrelidin). | **9** |
| **Figure S7:** Expanded ^1^H NMR spectrum, from 5.1-7.3 ppm (CDCl_3_, 400 MHz) of compound **2** (borrelidin). | **9** |
| **Figure S8:**^13^C NMR spectrum (CDCl_3_, 100 MHz) of compound **2** (borrelidin). | **10** |
| **Figure S9:** DEPT135 spectrum (CDCl_3_, 100 MHz) of compound **2** (borrelidin). | **10** |
| **Figure S10:** HSQC correlations of compound **2** (borrelidin). | **11** |
| **Figure S11:** HMBC correlations of compound **2** (borrelidin). | **11** |
| **Figure S12:**^1^H-NMR spectrum (DMSO-*d*_6_, 400 MHz) of compound **3** (1*H*-pyrrole-2-carboxylic acid). | **12** |
| **Figure S13:** APT spectrum (DMSO-*d*_6_, 100 MHz) of compound **3** (1*H*-pyrrole-2-carboxylic acid). | **12** |
| **Figure S14:** HMBC correlations of compound **3** (1*H*-pyrrole-2-carboxylic acid). | **13** |
| **Figure S15:** Mass spectrum of compound **1**(docosanoic acid). | **14** |
| **Figure S16:** Mass spectrum of compound **3** (1*H*-pyrrole-2-carboxylic acid). | **14** |

**Chromatographic investigation of the EtOAc extract of S17 isolate**

The ethyl acetate extract (700 mg) was applied to silica gel column chromatography (35 g), packed in CH_2_Cl_2_ 100% and eluted with CH_2_Cl_2_-MeOH mixtures with different polarities. Fractions (20 ml each) were collected and monitored using TLC. Fractions (16-19), 30 mg, eluted with CH_2_Cl_2_ (100%) afforded compound **1** (R*_f_* 0.65, 18 mg). Fractions (21-22), eluted with CH_2_Cl_2_- MeOH (98:2), afforded compound **2 (**R*_f_* 0.39, 7 mg). Fractions (26-41, 25 mg), eluted with the same polarity, were further purified using Sephadex LH20 column and CH_2_Cl_2_- MeOH (95:5 v/v). Fractions (5 ml each) were collected using silica gel TLC. Sub-fractions (24-30) afforded compound **3** (R*_f_* 0.25, 10 mg).

**Table S1. Composition of the supplied media**

| **Name** | **Composition** | |
| --- | --- | --- |
| **ISP2 medium** | | Yeast extract 0.4%, malt extract 1% and glucose 0.4%. |
| **Luria-Bertani broth** | | Tryptone1%, yeast extract 0.5%, and NaCl 1.0%. |
| **King A medium** | | Peptone 2%, K_2_SO_4_ 1%, and MgCl_2_ 0.14%. |
| **GSS medium** | | Starch 1%, glucose 2%, molasses 0.5%, yeast extract 0.5%, pepton 0.5% and CaCO_3_ 0.2%. |
| **GSM medium** | | Starch 1%, glucose2%, soybean meal 2.5%, beef extract 0.1%, Yeast extract 0.4%, NaCl 0.2%, K2HPO4 0.025%, and CaCO3 0.2% |
| **M2 medium** | | Glucose 1%, yeast extract 0.1%, meat extract 0.4%, peptone 0.4% and NaCl 0.2% |

**Table S2. Distribution of *Streptomyces* isolates purified from different localities**.

| **Location** | **Number and type of soil samples** | **Streptomyces characters** | | | |  |
| --- | --- | --- | --- | --- | --- | --- |
|  |  | **White** | **Grey** | **Yellow** | **Red** | **Total** |
| **1.Dakahlia** | 8/Cultivated | 16 | 19 | 4 | 2 | 41 |
| **2.Damietta** | 2/Sandy | 7 | 11 | 1 | 0 | 19 |
| **3.Cairo** | 3/Cultivated | 3 | 0 | 0 | 0 | 3 |
| **4.Suez** | 3/Sandy | 2 | 0 | 0 | 0 | 2 |
| **Total** | | 28 | 30 | 5 | 2 | 65 |

**Figure S1**.A) Assay of quorum sensing inhibiting activity of the tested *Streptomyces* isolates using *C. violaceum* CV026, isolates S17 and S12 showed early QSI effects but S6 showed lower yield with delayed production (*; *p* <0.05). **B**) Quantitative bioactivity of the cell free supernatant, ethyl acetate extract from S17, proteinase K treated extract, heated extract compared to solvent control(**; *p* <0.001).

**NMR Spectra of compounds 1-3:**

**
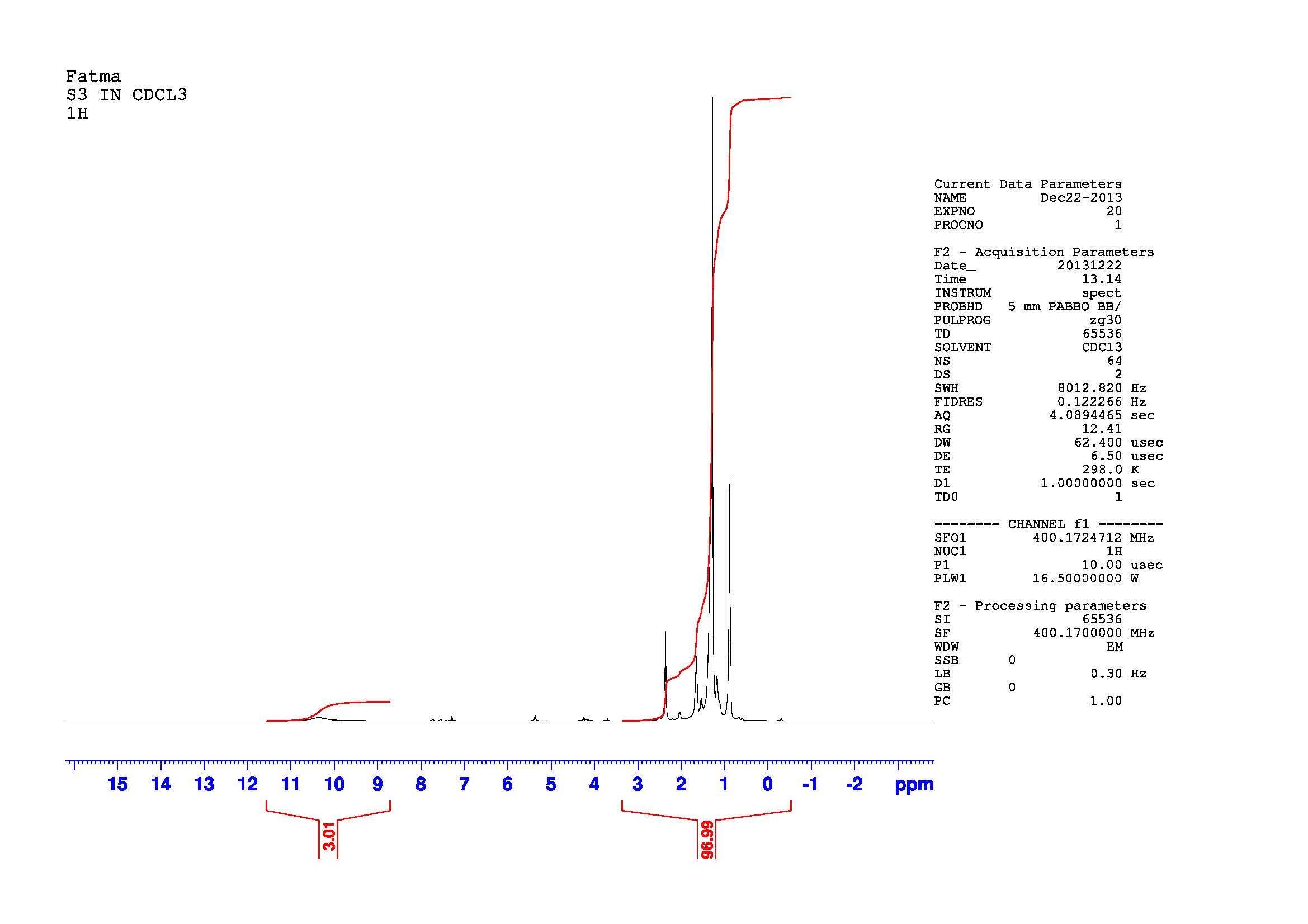
**

**Figure S2:**^1^HNMR spectrum (CDCl_3_, 400 MHz) of compound **1**behenic acid (docosanoic acid).


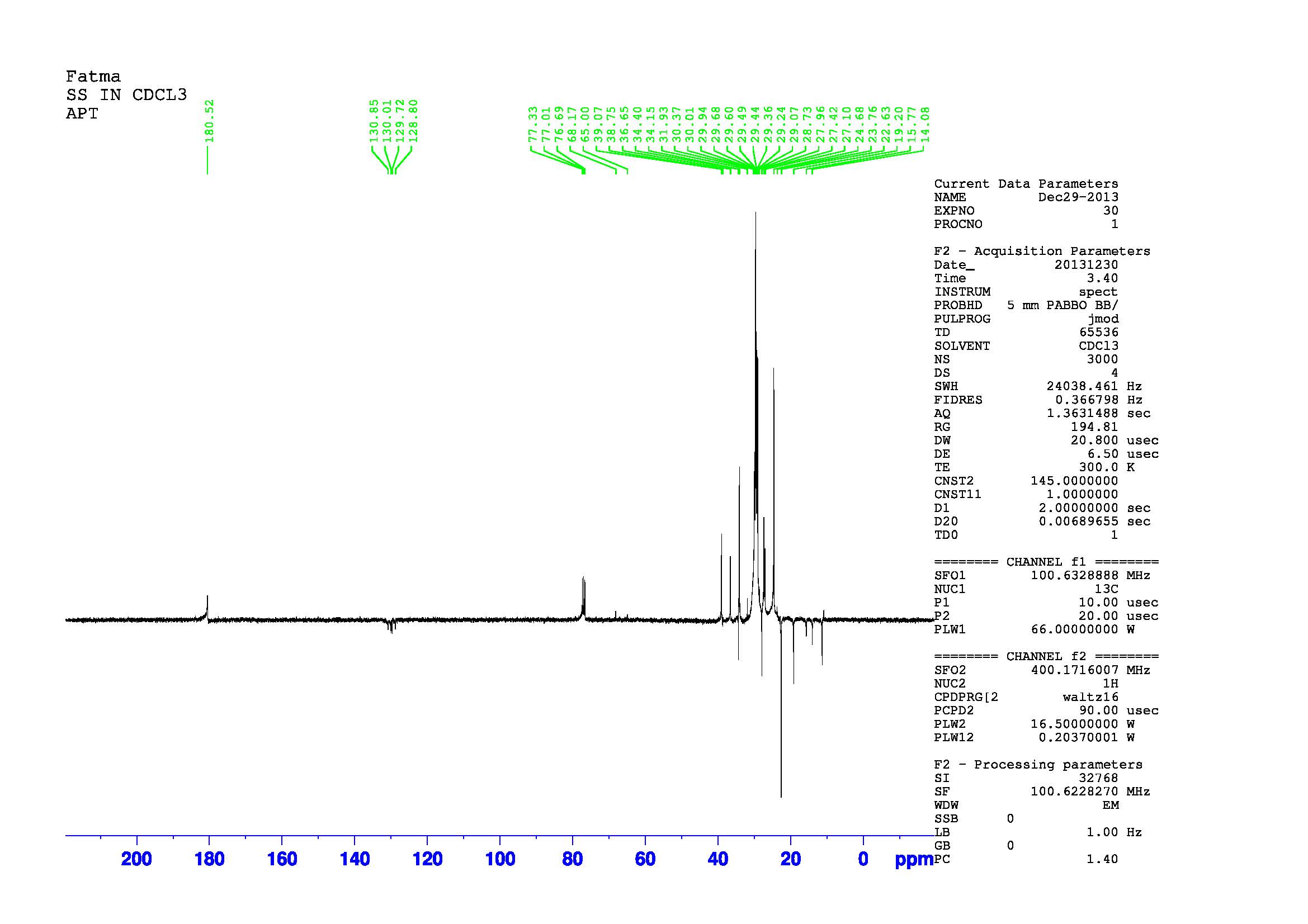

**Figure S3:** APT spectrum (CDCl_3_, 100 MHz) of compound **1**behenic acid (docosanoic acid).


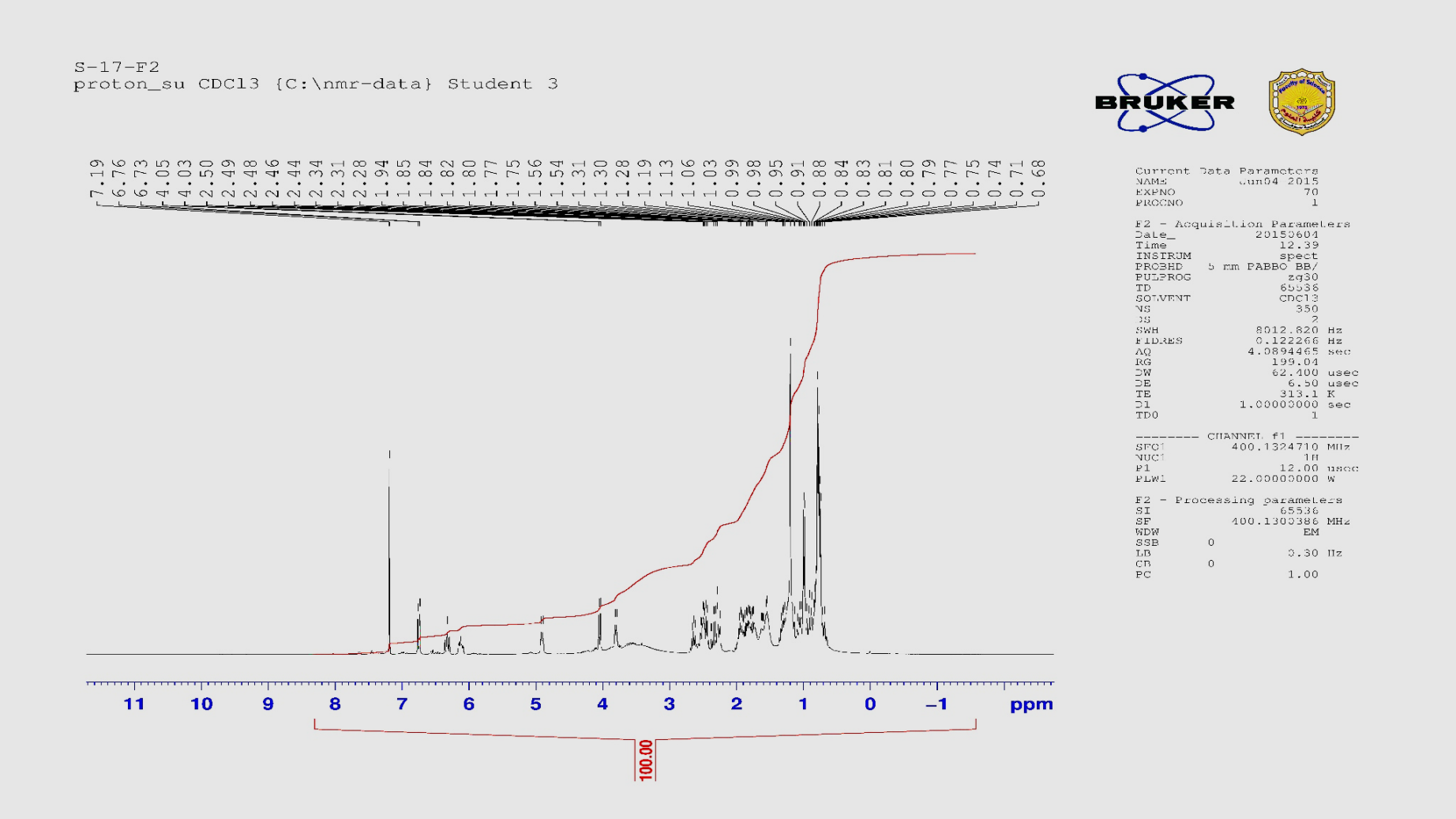

**Figure S4:**^1^H NMR spectrum (CDCl_3_, 400 MHz) of compound **2** (borrelidin).


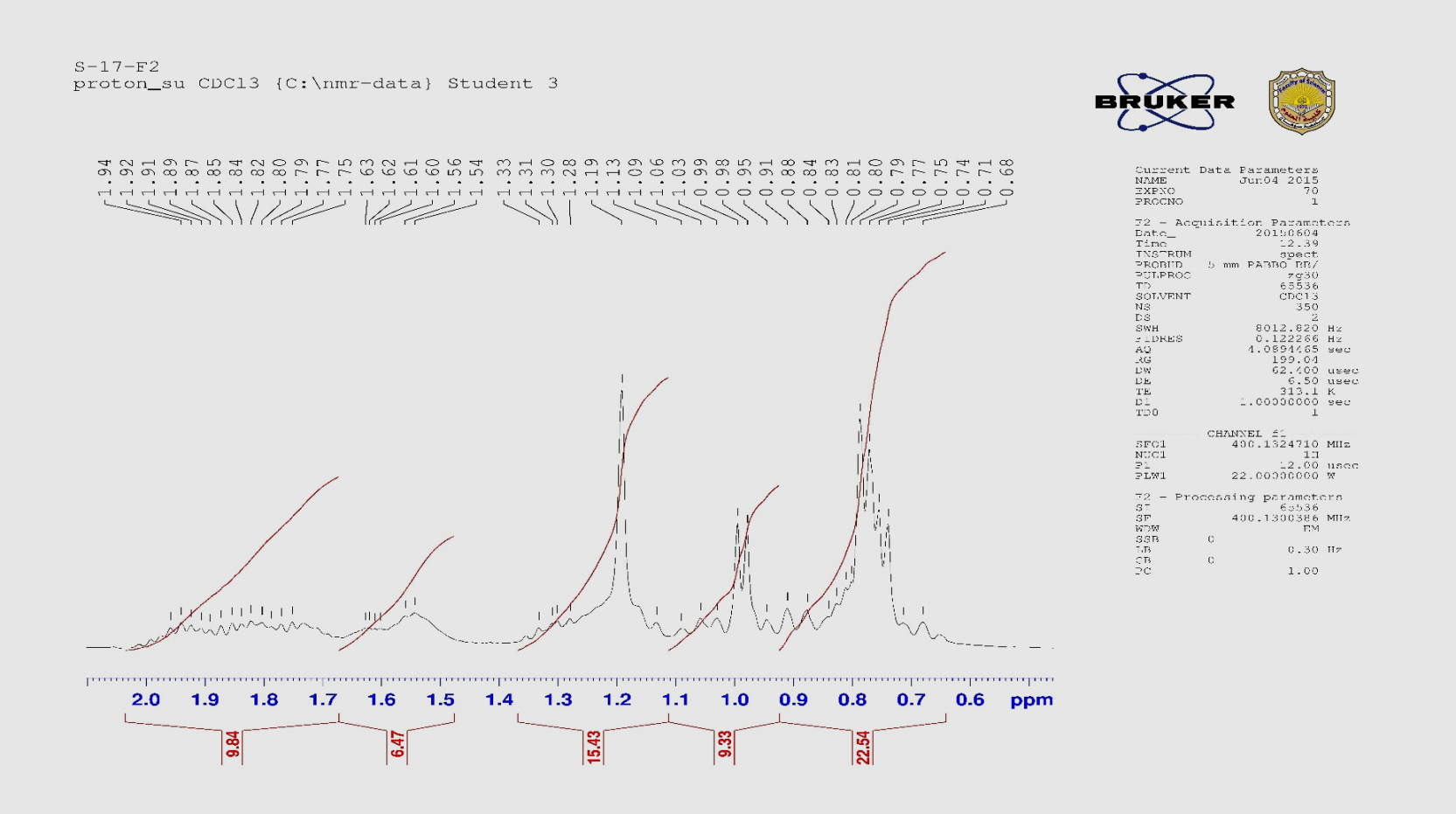

**Figure S5:** Expanded ^1^H NMR spectrum, from 0.6-2.0 ppm (CDCl_3_, 400 MHz) of compound **2** (borrelidin).

.
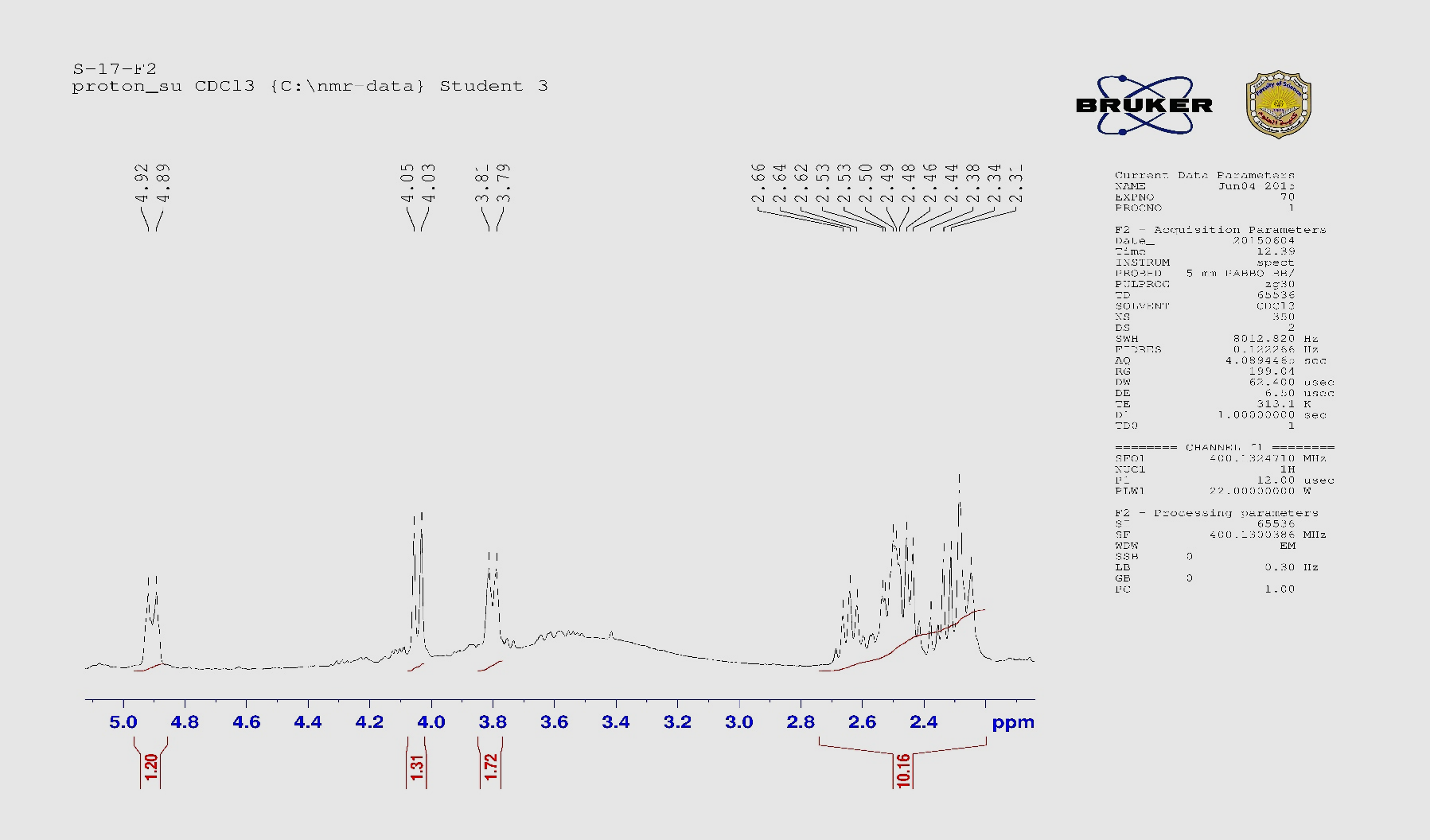

**Figure S6:** Expanded ^1^H NMR spectrum, from 2.0-5.1 ppm (CDCl_3_, 400 MHz) of compound **2**(borrelidin).


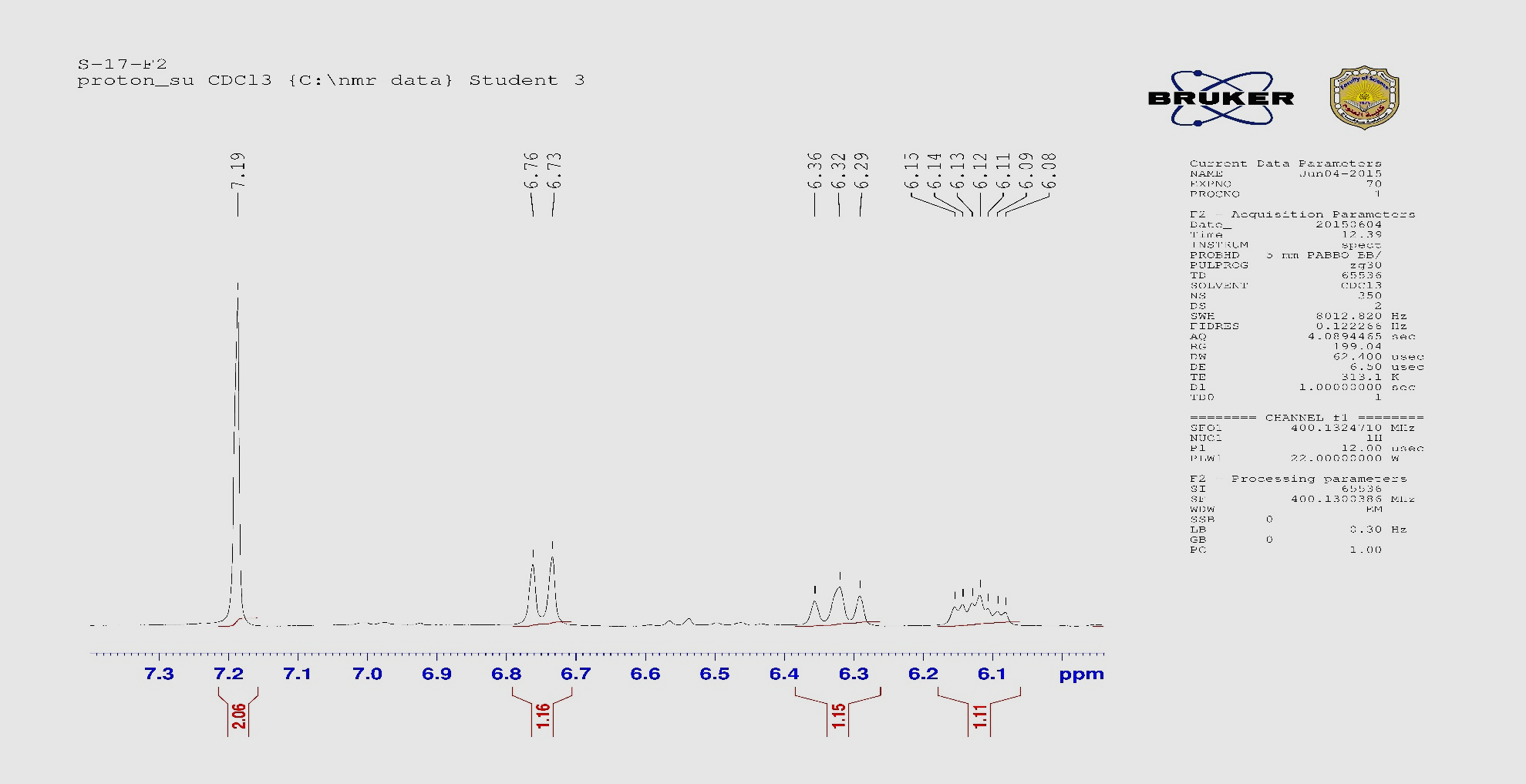

**Figure S7:** Expanded ^1^H NMR spectrum, from 5.1-7.3 ppm (CDCl_3_, 400 MHz) of compound **2**(borrelidin).


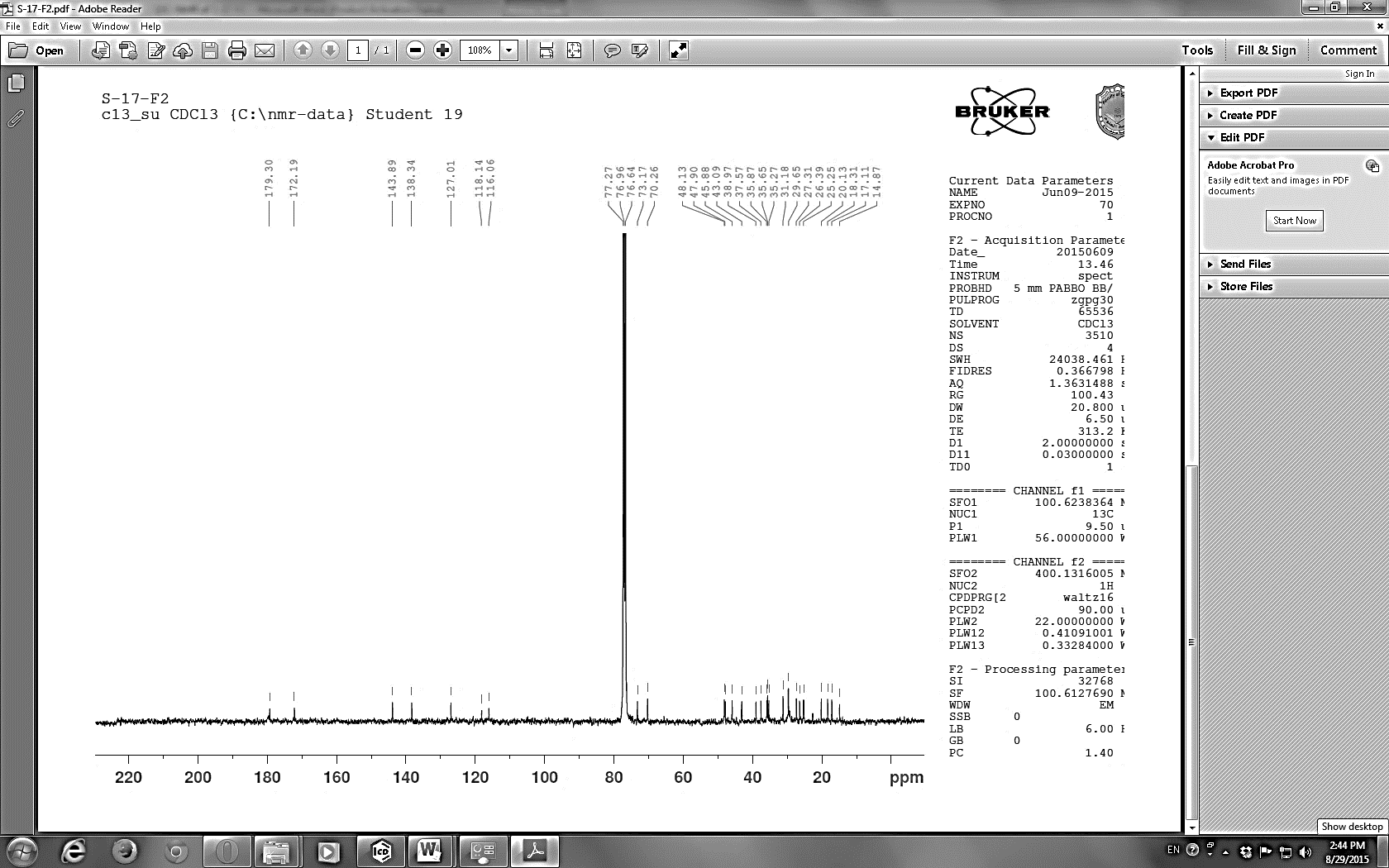

**Figure S8:**^13^C NMR spectrum (CDCl_3_, 100 MHz) of compound **2** (borrelidin).


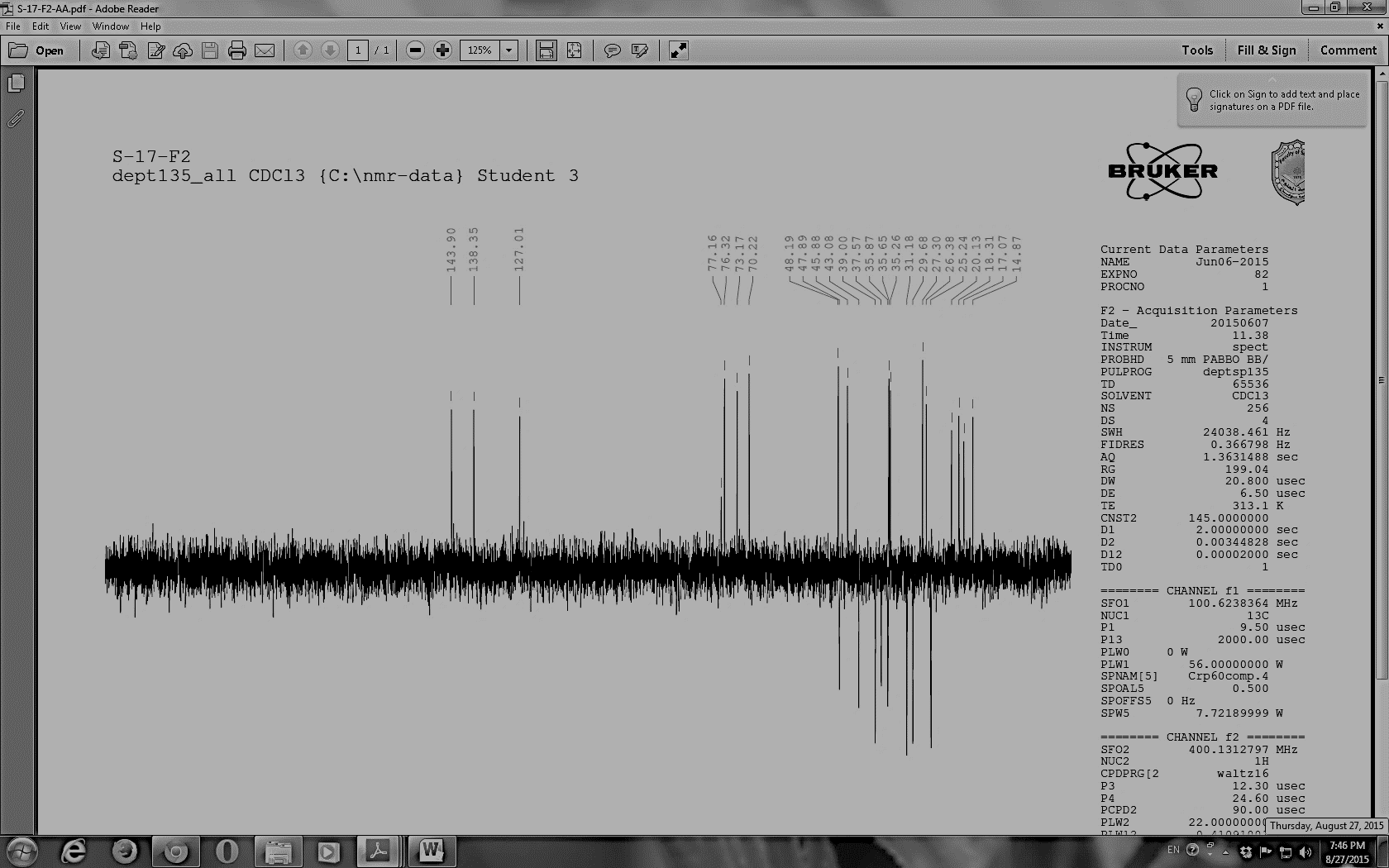

**Figure S9:** DEPT135 spectrum (CDCl_3_, 100 MHz) of compound **2**(borrelidin).


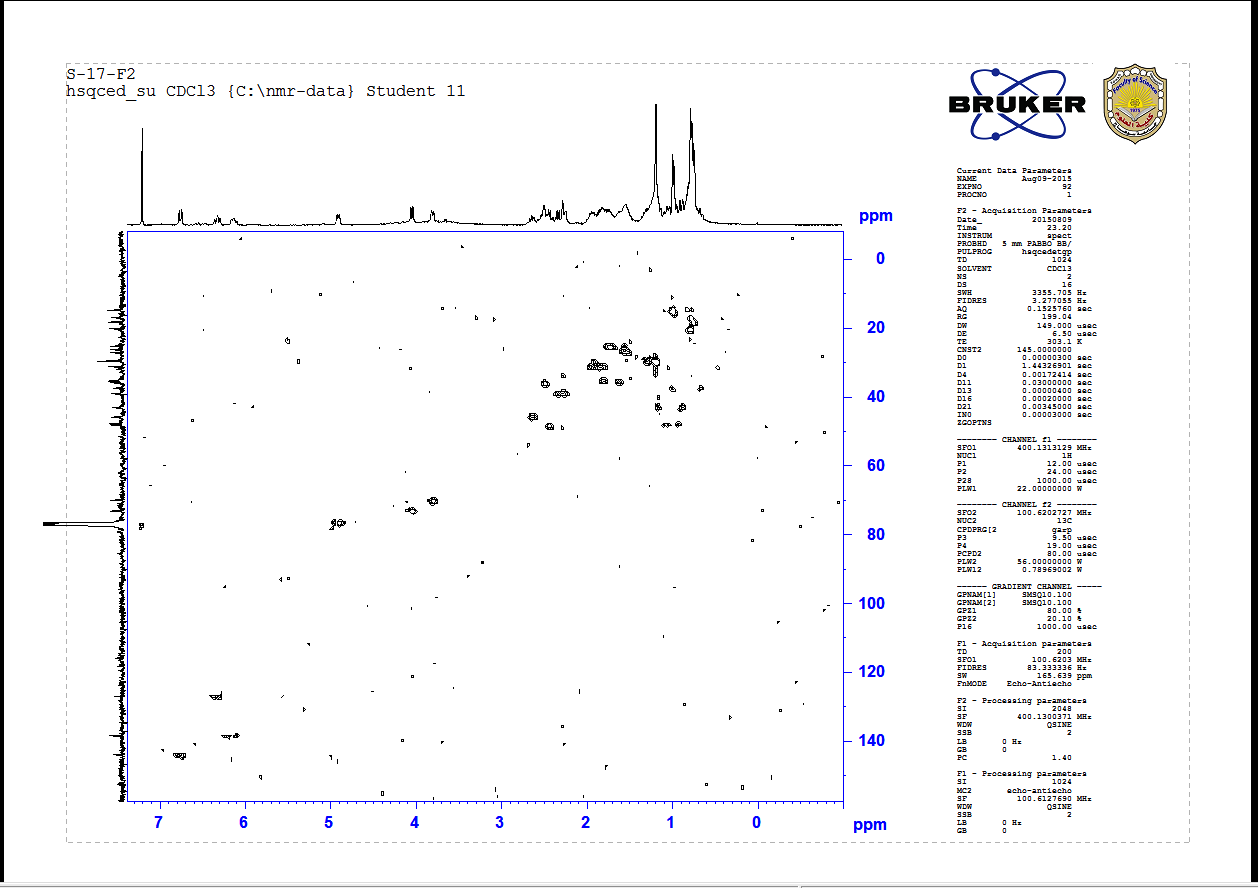

**Figure S10:** HSQC correlations of compound **2**(borrelidin).


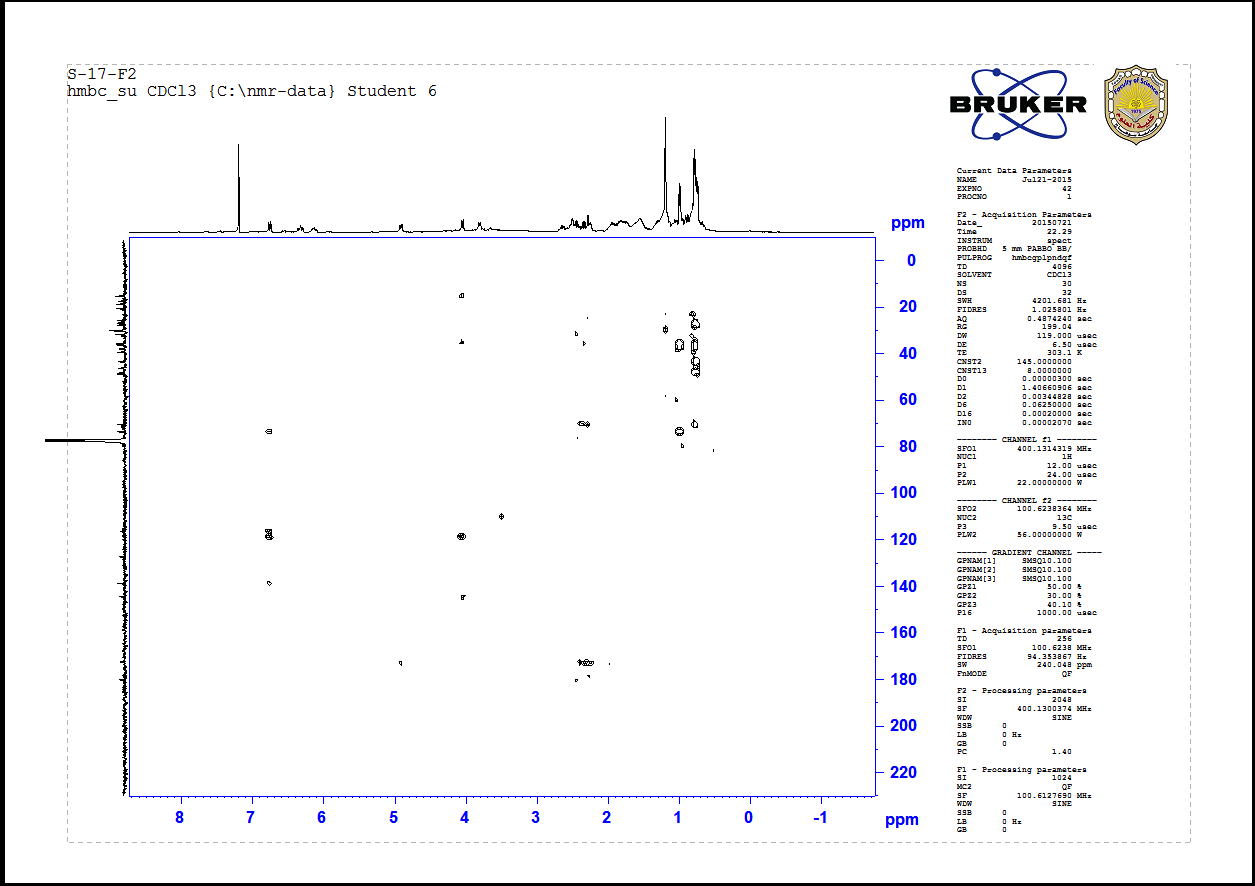

**Figure S11:** HMBC correlations of compound **2**(borrelidin).


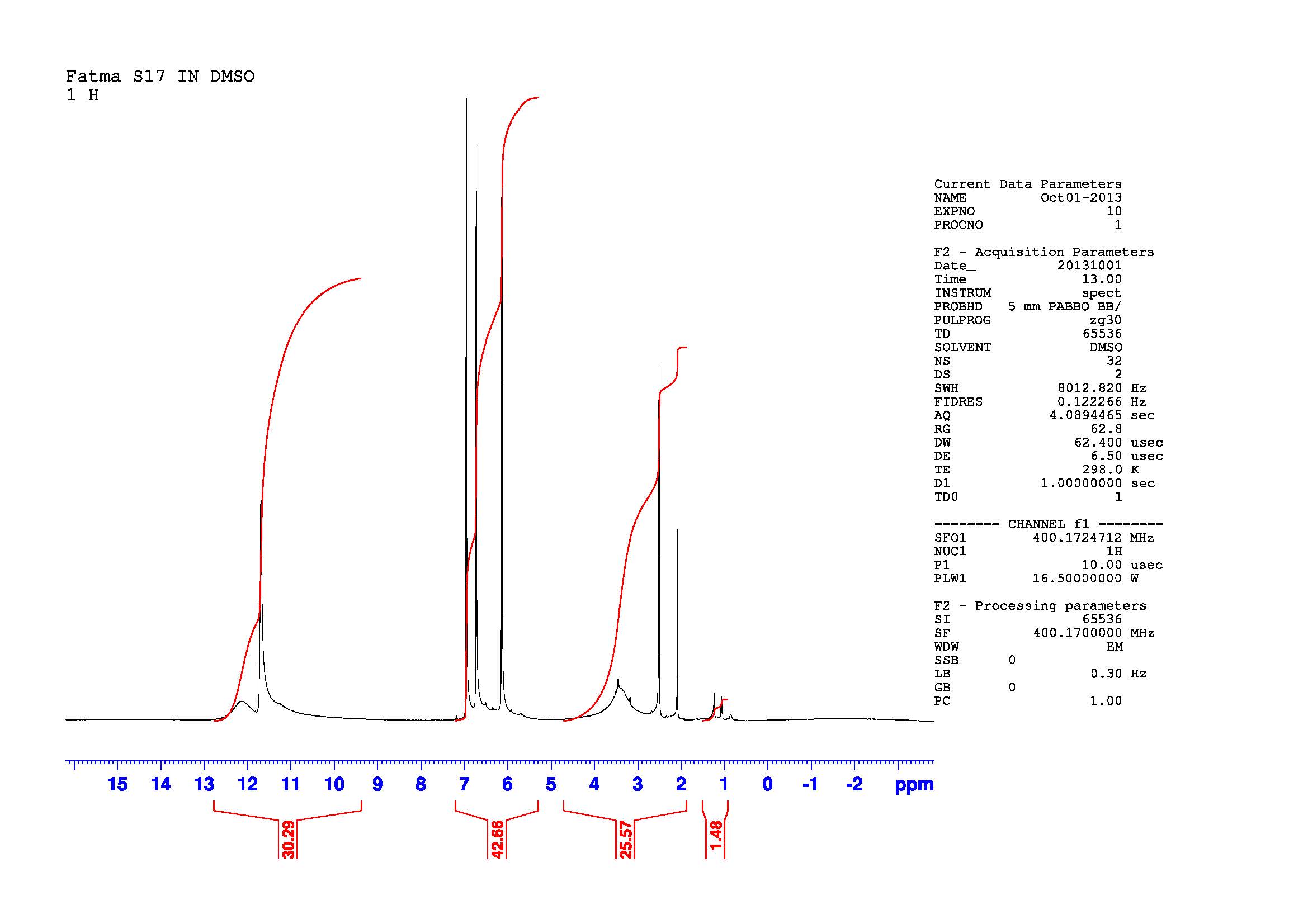

**Figure S12:**^1^H-NMR spectrum (DMSO-*d*_6_, 400 MHz) of compound **3**(1*H*-pyrrole-2-carboxylic acid).


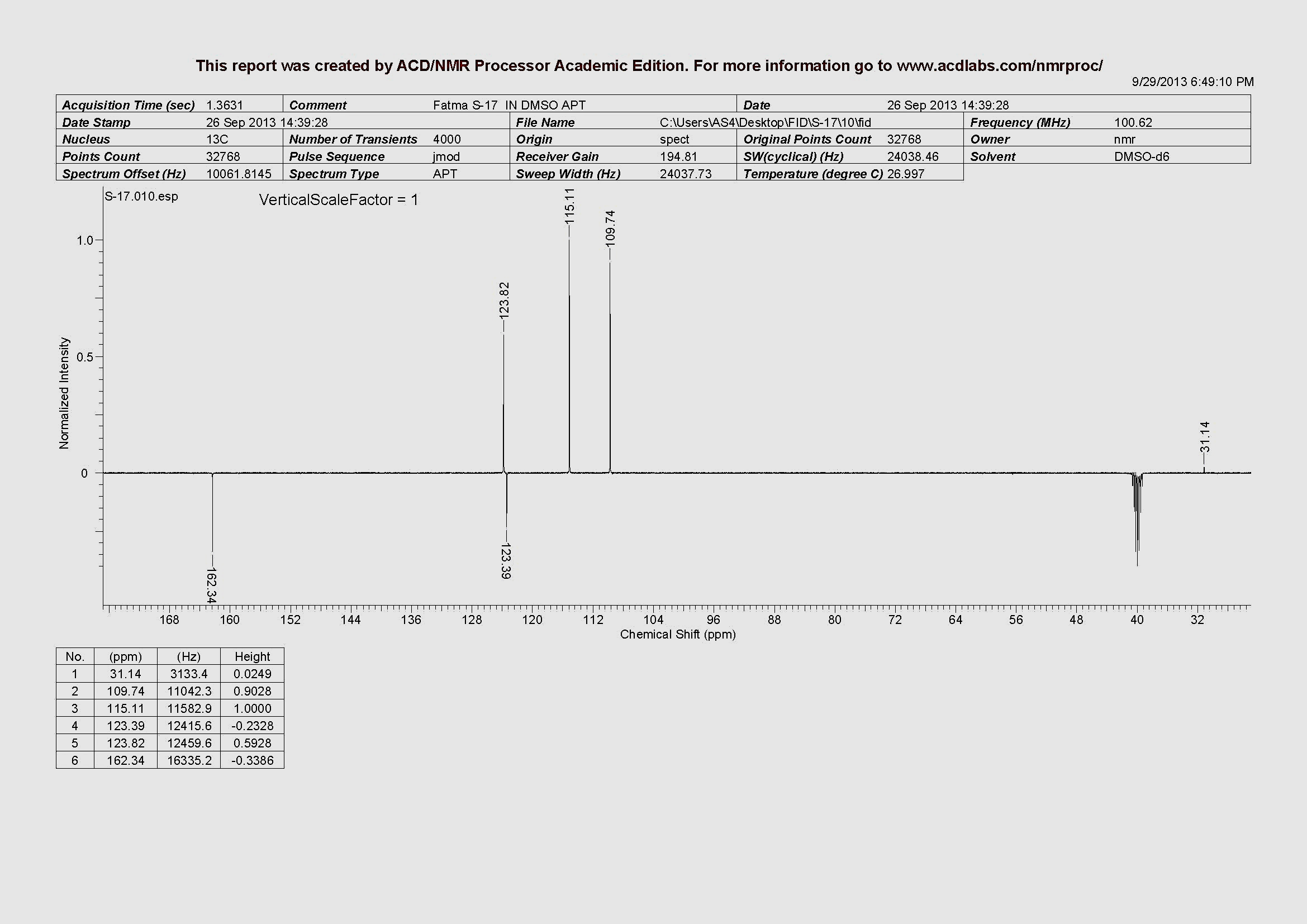

**Figure S13:** APT spectrum (DMSO-*d*_6_, 100 MHz) of compound **3** (1*H*-pyrrole-2-carboxylic acid).


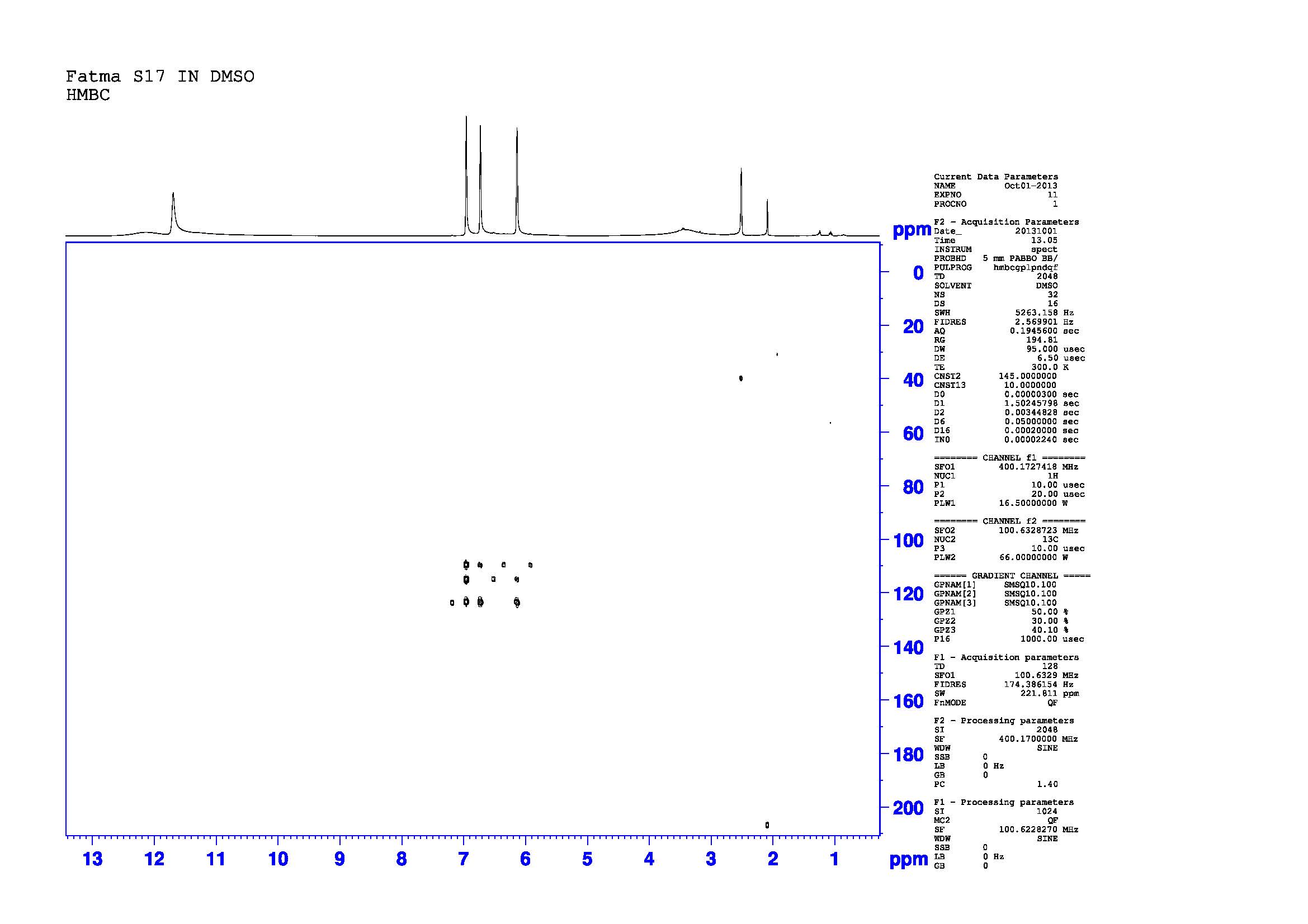

**Figure S14:**HMBC correlations of compound **3** (1*H*-pyrrole-2-carboxylic acid).

**Figure S15:** Mass spectrumof compound **1**(docosanoic acid).

**Figure S16:** Mass spectrum of compound **3** (1*H*-pyrrole-2-carboxylic acid).
